# Electrochemical synthesis of biobased polymers and polymer building blocks from vanillin†

**DOI:** 10.1039/d1ra00649e

**Published:** 2021-03-01

**Authors:** Robin Kunkel, Volkmar M. Schmidt, Carsten Cremers, Dominik Müller, Detlef Schmiedl, Jens Tübke

**Affiliations:** Fraunhofer Institute for Chemical Technology ICT, Department of Applied Electrochemistry Joseph-von-Fraunhofer-Str. 7 D-76327 Pfinztal Germany robin.kunkel@ict.fraunhofer.de; Mannheim University of Applied Sciences, Institute of Chemical Process Engineering Paul-Wittsack-Str. 10 D-68163 Mannheim Germany; Fraunhofer Institute for Chemical Technology ICT, Department of Environmental Engineering Joseph-von-Fraunhofer-Str. 7 D-76327 Pfinztal Germany

## Abstract

Vanillin, one of the few biobased aromatic compounds available on an industrial level, is an attractive candidate for the synthesis of biobased polymers and polymer building blocks. This study presents a detailed investigation of the reductive electrochemical coupling process by pinacolization of vanillin and divanillin in an electrochemical H-type cell setup to the polymer building block hydrovanilloin and to polyvanillin, respectively. Therein, different cathode materials are screened by linear sweep voltammetry for their capability and activity of hydrodimerization of phenolic aromatic aldehydes in alkaline aqueous media. Product distributions and faradaic efficiencies of the electrochemical vanillin reduction are investigated in bulk electrolysis experiments. Dependencies on electrochemical parameters such as current densities, applied charges and cathode materials are studied. Furthermore, the polyvanillin synthesis from divanillin is also investigated by bulk electrolysis experiments. The effects of selected electrochemical parameters (current density, applied charge and electrode material) on yield and structural features (weight-average molecular weight (*M*_W_), number-average molecular weight (*M*_N_), polydispersity (*M*_W_/*M*_N_)) measured by size exclusion chromatography of the obtained polyvanillin were evaluated. Structural features of isolated polyvanillin were determined by 2D-NMR (HSQC, ^13^C/^1^H) analyses and by ^31^P-NMR analyses after *in situ* labeling with Cl-TMDP and possible pathways for their generation are discussed. These two promising electro-synthetic processes studied are free of hazardous materials and reagents and highlight the contributions of preparative electrochemistry to green chemistry and further pave the way toward the application of electrochemistry in the synthesis of biobased building blocks and polymers.

## Abstract 2

Hydrovanilloin and polyvanillin were synthesized electrochemically investigating the impact of process parameters such as electrode material, charge and current density.

## Graphical abstract

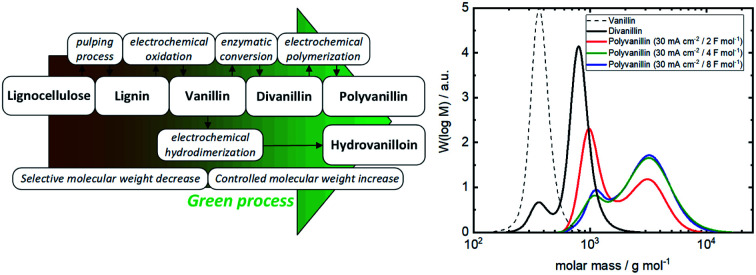

## 1 Introduction

The development of sustainable processes exploiting renewable resources for the production of new green monomers, drop-in chemicals replacing petrochemicals, and biobased polymers is of urgent matter.^1–4^ Therefore, several approaches have been recently published valorizing lignin, which is obtained on a million-ton scale from lignocellulose by either the Kraft process, the sulfite process or the organosolv process in the paper and pulp industry. Currently lignin is mostly used energetically to provide process heat and for the recovery of the pulping chemicals being the least utilized lignocellulosic biopolymer.^5–7^ One promising valorization route is the production of the fine chemical vanillin, which is generated on an industrial scale with appropriate yields up to 7% and high selectivity *via* classical thermal oxidation by oxygen at copper catalysts^8^ or more recently by sustainable electrochemical oxidation.^9–11^ Due to vanillin's aromatic character, multi-functionality and abundance, many attempts have been made to utilize vanillin. For example, over the last years vanillin has been intensively studied as key-intermediate in high performance biobased polymer synthesis, which was reviewed in 2015 by Fache *et al.*^12^ Vanillin has been exploited as a building block in, *e.g.* phenolic polymers, epoxy and benzoxazine resins, polyesters as well as acrylate and methacrylate polymers.

Electrochemical conversion of vanillin would be an attractive route enabling an overall green process, since electrochemistry is intrinsically fulfilling several important criteria of green chemistry, such as prevention of waste and use of toxic or hazardous reagents as only electrons serve as reactants. Moreover, this leads to a better atom efficiency and an inherently safe process. Also, electrochemical conversions often can be conducted under mild conditions, whereas for comparable conventional syntheses high temperatures and pressures are needed.^13,14^ Despite the mentioned advantages electrochemistry is nowadays still sparsely used as synthetic process step in the preparative organic chemistry.^15,16^

**Scheme 1:**
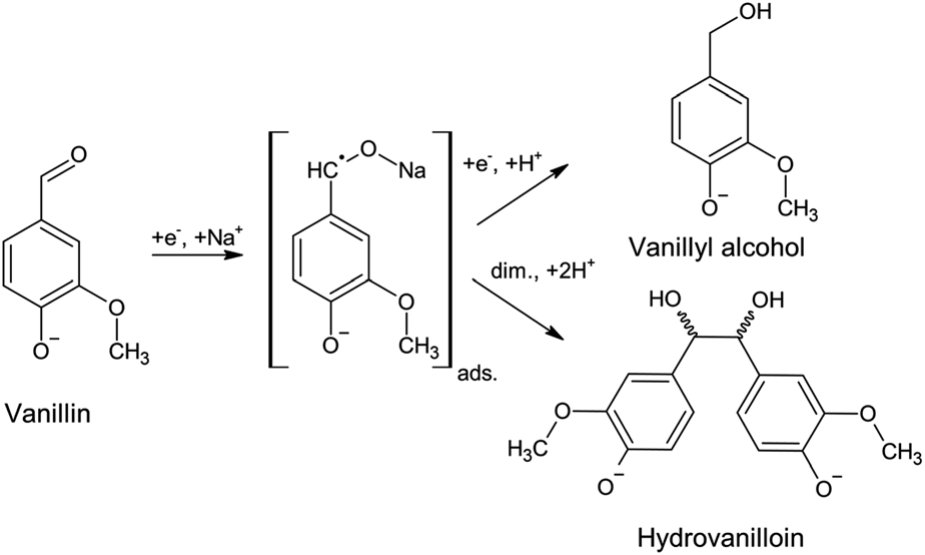
Electrochemical vanillin reduction in alkaline aqueous sodium hydroxide solution. The covalent bond O–Na of the metal ketyl is only used for convenience.^18^

A promising first reaction step for the synthesis of biobased thermoplastic, such as polycarbonates and thermosetting resins, is the electrochemical conversion of vanillin to its hydrodimer hydrovanilloin by pinacolization of the aldehyde group. Electrochemical pinacolization of vanillin to hydrovanilloin was first described in 1952 by I. A. Pearl at lead electrodes in aqueous sodium hydroxide solution.^17^ Jow *et al.* later investigated product distributions and the kinetics of the cathodic reduction of vanillin at a mercury pool cathode.^18^ Vanillyl alcohol and hydrovanilloin were found as the only products, in which the latter being the dominant product at lower current densities, higher pH values and high Na^+^ concentrations. On the basis of their results and published literature of the electroreduction of aromatic aldehydes they proposed the following mechanism, where vanillin is reduced *via* an adsorbed metal ketyl intermediate (Scheme 1).^19,20^ Due to the bisphenolic structure of hydrovanilloin the molecule could be used as a renewable drop-in for exemplary bisphenol A. The feasibility of the usage of hydrovanilloin as building block was shown *e.g.* in the synthesis of a hydrovanilloin-formaldehyde copolymer^21^ or a hydrovanilloin-diglycidyl ether phenoxy resin.^22^

**Scheme 2:**
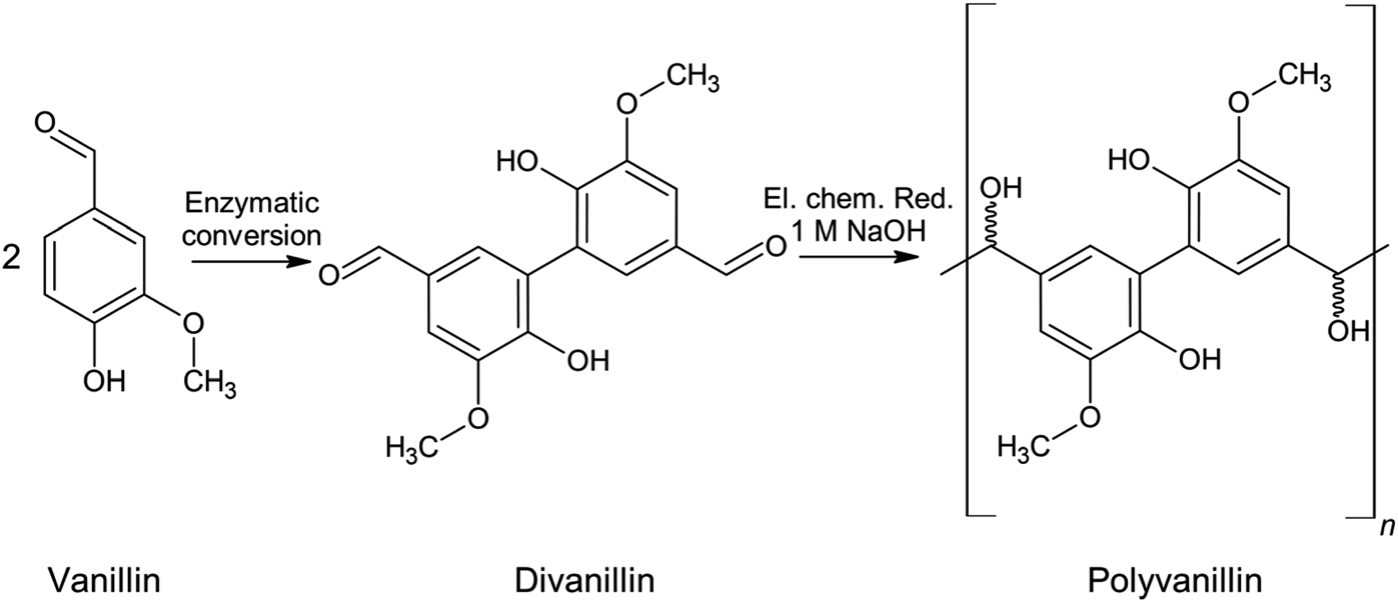
Synthesis route of vanillin to polyvanillin.

Another electrochemical approach exploiting the same reaction type of pinacolization is the synthesis of polyvanillin by reductive polymerization of 6,6′-dihydroxy-5,5′-dimethoxy-(1,1′-biphenyl)-3,3′-dicarboxaldehyde, commonly called divanillin or 5,5′-bis-vanillin (Scheme 2). The feasibility of this synthesis was shown by Amarasekara *et al.* in 2012 at a lead cathode in 1 M sodium hydroxide solution leading to a total vanillin-based polymer.^23^ The oxidative phenol coupling of vanillin to divanillin can be classically performed by means of Fe(ii)-based catalysts with yields of about 50%.^24–28^ The sustainable synthesis is performed enzymatically employing either laccase from Trametes versicolor in oxygenated solution^29^ or horse-radish peroxidase type I and hydrogen peroxide^30–33^ with yields of about 80% enabling a green alternative for this C–C coupling step. Besides for the electrochemical synthesis of polyvanillin divanillin was *e.g.* used in the preparation of Schiff-base polymers, which were investigated as chelating agents with metal ions.^34^

**Fig. 1:**
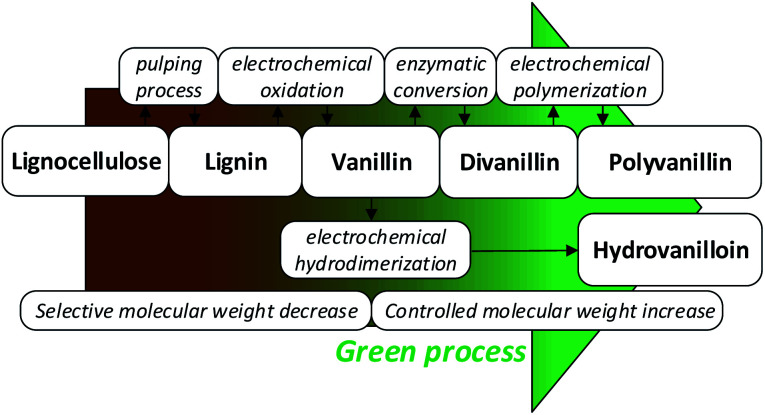
Sustainable value-added chain of lignocellulose to the biobased polymer polyvanillin and the polymer building block hydrovanilloin.

Fig. 1 summarizes the biobased value-added chain from lignocellulose to the biobased polymer polyvanillin and the polymer building block hydrovanilloin. As these product lines only employ sustainable process steps and the use of the side stream product lignin as starting material, a total green process would be realized.

The electrochemical reduction of the aldehyde groups in the pinacolization steps compete in aqueous alkaline media with the hydrogen evolution reaction (HER):

1 2H_2_O + 2e^−^ → 2OH^−^ + H_2_

Therefore, cathode materials showing sufficient high overpotentials for the HER are required to achieve adequate faradaic efficiencies (FEs) for the pinacol product. These are often hazardous materials such as lead, mercury and cadmium, which can suffer from cathodic corrosion due to dissolution or can form highly toxic organometallic compounds.^35,36^ The use of these heavy metals electrode materials, as in the above mentioned earlier studies, should therefore be avoided in future electro-synthetic processes.

To further establish reliable electrochemical processes for the pinacolization C–C coupling steps of vanillin to hydrovanilloin and divanillin to polyvanillin, a more detailed understanding of the underlying reaction mechanisms and the effects of electrochemical parameters, such as current densities, applied charges, on product distributions, yields and structural features are required. The present study addresses these subjects extending the feasibility study of polyvanillin and the state of the art of the electrochemical synthesis of hydrovanilloin as well as further screens for green, sustainable and stable electrode materials as alternatives for the commonly used heavy metals.

## 2 Experimental

### 2.1 Chemicals

All aqueous solutions were prepared with ultrapure water (0.055 μS cm^−1^). Vanillin (Sigma Aldrich, reagent plus, 99%), vanillyl alcohol (Sigma Aldrich, supelco, ≥99%), vanillic acid (Sigma Aldrich, purum, ≥98%), sodium hydroxide (Carl Roth, p.a., ≥98%), 1 M hydrochloric acid (Carl Roth, standard solution/p.a.), concentrated hydrochloric acid (VWR Chemicals, supelco), methanol (Carl Roth, LC-MS grade), ethanol absolute (VWR Chemicals, LC-MS grade), acetic acid (Carl Roth, Ph. Eur., 100%), H_2_O_2_ solution (Sigma Aldrich, Supelco, ≥30%), horseradish peroxidase (Sigma Aldrich, Type I, ≥50 units per mg), acetonitrile (Sigma Aldrich, HPLC grade), *ortho*-phosphoric acid (Carl Roth, Ph. Eur., 85%), DMSO-d_6_ (Sigma Aldrich, 99.9 atom% D), pyridine-d_5_ (Sigma Aldrich, for NMR spectroscopy, min. 99.8% D), CDCl_3_ (Sigma Aldrich, for NMR spectroscopy, min. 99.9% D), 2-chloro-4,4,5,5-tetramethyl-1,3,2-dioxaphospholane also called Cl-TMDP (Sigma Aldrich, 95%, for *in situ* labelling in ^31^P-NMR-analysis), cyclohexanol (Sigma Aldrich, 99%, internal standard) were used as received for all experiments. As electrode materials Pb sheets (ChemPur, 99.99%), Zn sheets (ChemPur, 99.99%), glassy carbon (GC – HTW Germany, SIGRADUR®) and Ni-foam (AQUA-TITAN, Germany) were used.

### 2.2 Electrochemical experiments

#### 2.2.1 Set-up

**Fig. 2:**
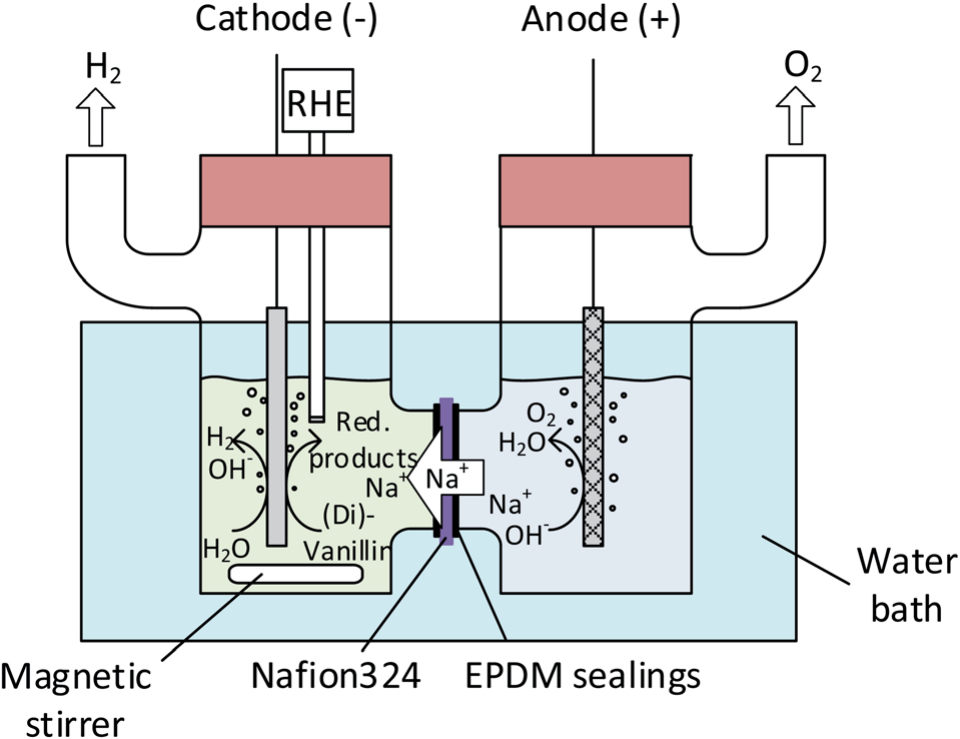
Electrochemical batch cell set-up (H-type).

A conventional electrochemical cell (H-type) with a standard three electrode setup was built for linear voltammetry studies as well as for bulk electrolysis experiments (Fig. 2). Anode and cathode compartments were separated by a Nafion® N324 membrane (Ion Power GmbH), which was brought to the Na^+^-form by soaking it in 1 M NaOH for at least 24 h. A Bio-Logic SAS SP-150 potentiostat and a VMP3 10 A booster were used in all electrochemical experiments. The catholyte was stirred by a magnetic stirrer at 250 rpm throughout all bulk electrolysis experiments. The H-type cell was immersed in a water bath and cell temperatures were confirmed to stay between 22–25 °C during all measurements.

#### 2.2.2 Electrodes

Three different working electrodes consisting of Pb, Zn and glassy carbon (GC) sheets with dimensions of 2 × 6 cm^2^ were used. The geometric area immersed in the electrolyte facing the counter electrode was 5 cm^2^, which was used as reference area for the calculations of the current densities. To ensure no limitation by the parallel running reaction at the anode side, which is the oxygen evolution reaction (OER):

2 4OH^−^ → O_2_ + 2H_2_O + 4e^−^

a 2 × 2.5 cm^2^ Ni-foam anode was used in all experiments due to its fast kinetics of the OER and high surface area.^37^ Potentials were measured against a reversible hydrogen reference electrode (RHE – HydroFlex®, Gaskatel, Germany) placed as close as possible to the working electrode. The Ni-foam anode was used without preparation. Prior to each experiment in case of Pb and Zn the cathodes were polished with SiC papers with decreasing roughness (Struers GmbH, FEPA #P180/#P500/#P1000 – grade), rinsed with ultrapure water, dipped in absolute ethanol to remove organic impurities, held 5 s in 1 M HCl for Zn or concentrated HNO_3_ for Pb, respectively, and rinsed with ultrapure water again. GC electrodes were obtained mirror finished. The electrodes were roughly rinsed with water and absolute ethanol. When reusing them, the mirror finish was refreshed by polishing with diamond suspension with decreasing particle size (3 μm, 0.25 μm, 0.05 μm, Buehler, Germany). Mass changes of working electrodes were determined by weighing before and after the electrolysis experiments.

#### 2.2.3 Electrochemical procedures

For the vanillin reduction experiments the catholyte was prepared as 0.2 M vanillin solution by dissolving 1.522 g of vanillin in 50 mL of 1 M sodium hydroxide solution. For the divanillin reduction to polyvanillin a 0.1 M divanillin solution was prepared by dissolving 1.511 g of divanillin in 50 mL of 1 M sodium hydroxide. The anolyte consisted of 50 mL of 1 M sodium hydroxide solution in both cases. Immediately after the electrodes were immersed into the electrolyte and the setup was ready, the experiment's current was applied to the cell to minimize open-circuit corrosion of the cathode's surface in aqueous alkaline media. Linear sweep voltammograms (LSV) were recorded by sweeping the potential with a potential sweep rate of 10 mV s^−1^ from −0.5 V *vs.* RHE to −1.6 V *vs.* RHE. For measuring background currents, the same experiment was conducted without adding vanillin to the catholyte compartment. Electrolysis was performed in galvanostatic mode until a given charge had passed. Values of charges are presented in F per mole vanillin or divanillin, respectively, where F is the Faraday unit of charge, *i.e.* the charge of one mole of electrons (96 485 C). Fresh electrolyte solutions were prepared for each experiment. Recorded potentials were corrected by the solution's resistance between the working electrode and the reference electrode, which was measured by galvano electrochemical impedance spectroscopy (GEIS) prior to and after each experiment. Frequencies were measured from 100 kHz to 100 mHz at the experiment's electrolysis current. The alternating current amplitude was set to 10% of the electrolysis current. The vanillin electrolysis was monitored throughout electrolysis by withdrawing aliquots of 10 μL from the catholyte solution every 15–30 minutes depending on the overall reaction time. The aliquots were diluted with 100 μL methanol and 10 μL internal standard (vanillic acid) and then quantitively analyzed by capillary electrophoresis (CE) coupled with UV detection. FEs for the vanillin reduction products were calculated as follows:3
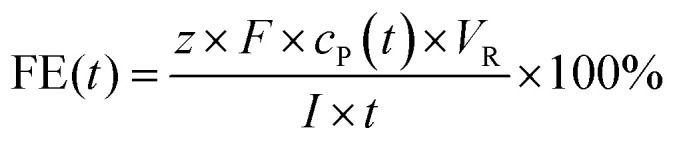
where *z* is the number of transferred electrons for a given product, *F* the Faraday constant (96 485 C mol^−1^), *c*_P_(*t*) the concentration of a product at a given time *t*, *V*_R_ the catholyte's volume and *I* the circuit current. The number of transferred electrons *z* is equal to 2 for vanillyl alcohol as well as for hydrovanilloin (see Scheme 1). The change of volume of the catholyte due to the withdrawing of the aliquots for the CE analysis was neglectable.

#### 2.2.4 Product isolation

The products were isolated after the electrolysis from the catholytes for both reaction as described in the literature.^23^ Therefore, the catholyte was transferred to a beaker, cooled in ice and acidified to pH = 2. For the titration step 1 M HCl was used in case of the vanillin reduction experiments and concentrated HCl in case of the divanillin reduction experiments. The precipitates were filtered, thoroughly washed with water and dried with silica gel in a desiccator under vacuum overnight. Isolated yields were reported by weighing the dried samples.

### 2.3 Analytical instrumentation

#### 2.3.1 Capillary electrophoresis (CE)

A CE system with UV detection (Prince Technologies, PrinCE-C770 and PrinCE Next875 DAD) was used to analyze the withdrawn aliquots in the vanillin electrolysis. A fused silica tubing of 50 μm inner diameter and an effective length of 8.5 cm (short capillary application) was used as capillary (total length of capillary was 60 cm). The capillary was conditioned before every run for 2 min (2000 mbar) with background electrolyte. The wavelength of the UV detector was selected to be 283 nm according to the literature.^18^ For the background electrolyte 0.8 g of phosphoric acid were dissolved in 20 mL 1 M NaOH, 30 mL Water and 50 mL methanol. The conductivity of the buffer solution was 6.1 mS cm^−1^ and the pH value 12.2 at 20 °C. Linear calibration curves were obtained from peak areas for vanillin and vanillyl alcohol from pure substances. Hydrovanilloin was obtained after a series of treatments of the catholyte solution, which is described elsewhere.^23^ 10 μL of vanillic acid were added to each sample as internal standard resulting in an end concentration of 1.7 mM. The electrophoretic separation was carried out at −20 kV and 20 °C (corresponding current in the range of −60 to −70 μA). The injection parameters were 60 mbar for 0.1 min at 20 °C.

#### 2.3.2 HPLC-DAD-MSD

HPLC-DAD-MSD analysis of selected isolated products of the vanillin reduction experiments and of divanillin was realized on a series 1200 HPLC-System from Agilent Technologies (binary pump SL 600 bars, auto sampler SL, DAD SL, LC MS Ion Trap XCT plus Series 6330 with multi-mode ion source MMAPCI/APESI). The products were dissolved in methanol and analyzed on a Kinetex C8 HPLC-column (2.6 μm, 100 Å, 3.0 mm × 100 mm). The DAD wave length was set to 280 nm ± 5 nm with a ref. wave length of 550 nm ± 50 nm. The mobile phase consisted of A: acetic acid (0.05%ical) and B: acetonitrile (in case of isolated product from vanillin) or methanol (in case of divanillin) in gradient program. The flow rate was set to 0.5 mL min^−1^ and the oven temperature to 45 °C.

#### 2.3.3 Size exclusion chromatography (SEC) analysis

The structural features weight-average molecular weight (*M*_W_) and number-average molecular weight (*M*_N_) of the isolated polymer samples from the divanillin reduction experiments were determined by SEC using a HPLC-system with a refractive index detector (Agilent Technologies, LC 1200, RID cell temperature: 35 °C) with three columns (PSS MCX 10 μm, guard column, analytical columns 10 μ, 100 Å and 10 μ, 1000 Å) according to the published literature.^38^ 2–3 mg of the samples were dissolved in 1 mL of 0.1 M sodium hydroxide solution. The mobile phase was 0.1 M sodium hydroxide solution with a flow rate of 1 mL min^−1^ and an oven temperature of 35 °C. Conventional pullulan standards (342 g mol^−1^ to 805 000 g mol^−1^, PSS Mainz, Germany) were used for calibration. Therefore, molecular weights of samples are presented *versus* pullulan.

#### 2.3.4 NMR spectroscopy

^1^H-, ^13^C- and 2D-NMR spectra (HSQC, ^13^C/^1^H) of isolated products were recorded on a Bruker AVANCE spectrometer operating at 500 MHz. In case of the vanillin reduction experiments DMSO-d_6_ was used as solvent and in case of the divanillin reduction experiments pyridine-d_5_. Before dissolution the samples were additionally dried under vacuum. Chemical shifts for ^1^H-NMR and ^13^C-NMR are given in ppm downfield from TMS (*δ* = 0.00). The spectra were adjusted to the C–H cross coupling signals of pyridine-d_5_ (*δ*_H_ in ppm/*δ*_C_ in ppm: 7.220/123.87; 7.580/135.91; 8.740/150.35) and of DMSO-d_6_ (*δ*_H_ = 2.5 ppm/*δ*_C_ = 39.51 ppm), respectively. Generated 2D-NMR (HSQC) spectra were processed on a MestReNova software.

Further, ^31^P-NMR spectra were recorded of selected isolated polyvanillin samples of the divanillin reduction experiments after *in situ* labeling for quantification of aliphatic and aromatic OH-groups. Three solvents were used for the preparation of the polyvanillin samples. Solvent I was a homogenous mixture of 16 mL pyridine (water free) and 10 mL CDCl_3_ (water free). For the preparation of solvent II 10.0 mg of Cr(iii)acetyl acetonate were weighed into a HPLC vial and 2 mL of solvent I were added. The vial was caped and the Cr(iii) acetyl acetonate was dissolved and homogenized at room temperature by vortex mixing. For the preparation of solvent III 21.92 mg of internal standard cyclohexanol (99%ical) was accurately weighed in a HPLC-vial and exactly 2 mL of solvent I was added. The vial was caped and the solution was homogenized at room temperature by vortex mixing. The freshly prepared solvents were allowed to stay at room temperature. The polyvanillin samples were dried in a vacuum oven at 10 mbar and at a temperature of 50 °C for 48 h. Exactly 20.00 mg of the dried polyvanillin samples were weighed in HPLC vials. Then 800 μL of freshly prepared solvent I, 100 μL of freshly prepared solvent II and 100 μL of freshly prepared solvent III were added to the samples and the vials were caped. The samples were mixed by vortex mixing at room temperature. 10 min before the ^31^P-NMR-analysis 160 μL of *in situ* labeling reagent 2-chloro-4,4,5,5-tetramethyl-1,3,2-dioxaphospholane (Cl-TMDP) were added to the vials and homogenized again by vortex mixing at room temperature. Only after the addition of Cl-TMDP were the samples completely dissolved and no precipitation was observed. The final sample solution was transferred to an NMR tube. The ^31^P-NMR analysis was done on a Bruker AVANCE 300 MHz. Chemical shifts are reported relative to the sharp signal (132.2 ppm) originating from the reaction between water and Cl-TMDP. Following NMR parameters were used: scans = 1024, pulse delay = 5 s, 90° pulse and line broadening = 2 and default baseline correction. ^31^P-NMR measurement is based on the developed method by Granata and Argyropoulos.^39^^31^P-NMR-spectra were processed on ACD Lab 12.0, 1D-processor software. Integration for quantitative calculation was done according to Pu *et al.*^40^ Integration limits for aliphatic OH-groups were 150.0–145.40 ppm, for phenolic OH groups 144.50–137.50 ppm and for the internal standard (cyclohexanol) 145.39–144.75 ppm (ESI, Fig. S12†).

### 2.4 Synthesis of divanillin

Divanillin was synthesized using horse radish peroxidase and hydrogen peroxide according to the procedure described in the literature.^41^ 10 g of vanillin were dissolved in 400 mL deionized water at a temperature of 40 °C by stirring. The pH value was adjusted to a value of 4 by adding 250 μL of glacial acetic acid. An amount of 1000 units of activity of horse radish peroxidase were added during stirring and 7.5 mL of 30 wt% hydrogen peroxide solution were added to the solution dropwise. The brown precipitate was filtered after 15 minutes, washed with hot water and dried under vacuum (10 mbar, at 45 °C, two days) (8 g, 80%). The product structure was confirmed by HPLC-DAD-MSD, elemental analysis (Thermo Scientific, Thermo Electron Flash EA 1112) and Fourier Transform Infrared analysis (Thermo Scientific, NICOLET 6700) (ESI, Fig. S1 and S2†). The remaining fraction of vanillin in the isolated divanillin batch was determined by GC-FID/MSD (Agilent Technologies, HP 6890) on a DB-5-ms column after calibration with vanillin standard solutions. The remaining fraction of vanillin was 4.9 wt% in the first batch and under limit of detection in the second.

## 3 Results and discussion

### 3.1 Screening of cathode materials for vanillin reduction

**Fig. 3:**
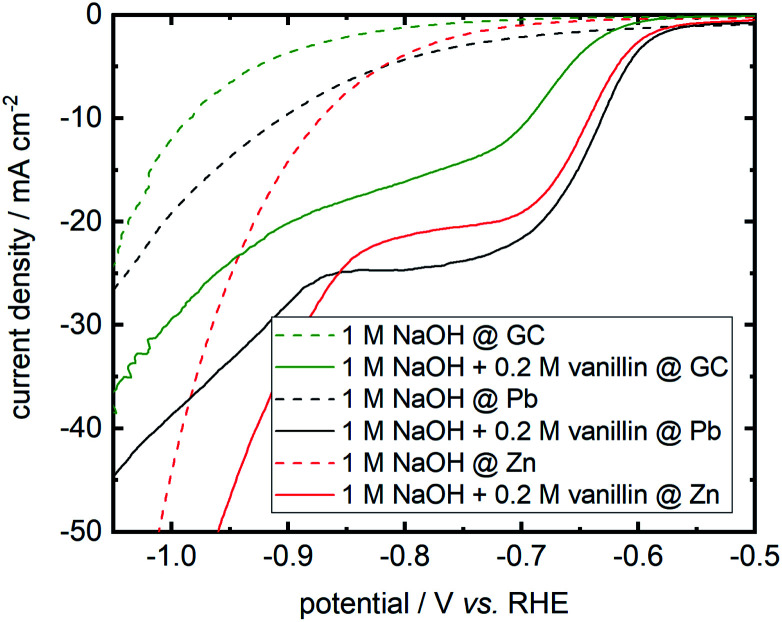
Cathodic LSVs for vanillin reduction at Pb, Zn and GC in static solution. Potential sweep rate is 10 mV s^−1^. Background currents of neat electrolyte are shown as dashed lines.

On repeating I. A. Pearls original synthesis^17^ of hydrovanilloin by electrochemical pinacolization of vanillin at lead cathodes, Pb dissolution was found in electrolysis under given conditions. As Zn and GC exhibit high overpotentials for the competing HER (see eqn (1)) and are cheap as well as harmless materials, they are screened for the electrochemical reduction of vanillin in alkaline aqueous media. Polarization curves of background currents resulting from the competing HER and of the actual vanillin reduction measured by LSV are shown in Fig. 3.

Background currents are neglectable for all three electrode materials at lower overpotentials up to −0.8 V *vs.* RHE. With increasing overpotential a significant increase of the HER currents are observed for the three investigated cathode materials. Background currents are then in order Zn followed by Pb and GC. For example, at a potential of −1 V *vs.* RHE currents of −44.3 mA cm^−2^ for Zn, −19.2 mA cm^−2^ for Pb and −12.2 mA cm^−2^ for GC are measured for the HER. At even more negative values background currents of Pb and GC are approaching each other, whereas these negative potentials are hardly achieved at Zn due the higher HER activity depolarizing the Zn cathode. All three cathode materials show activity for the electrochemical reduction of vanillin. Onset potential at Zn and Pb differ in the millivolt range for the electrochemical vanillin reduction at ≈−0.575 V *vs.* RHE. At GC the onset potential is shifted by 50 mV in negative direction and cathodic currents are inferior to Pb and Zn. At a potential of −0.800 V *vs.* RHE reductive currents of −24.7 mA cm^−2^ for Pb, −21.4 mA cm^−2^ for Zn and −16.1 mA cm^−2^ for GC are observed in static solution. Therefore, GC shows comparable the worst catalytic properties for the electrochemical vanillin reduction under the three screened electrode materials but exhibits the lowest activity for the competing HER. Almost similar catalytic properties to the commonly used Pb cathodes are observed for the electrochemical vanillin reduction at Zn cathodes, but also showing a higher activity of the competing HER in the higher current density region. To evaluate further the impact of the competing HER quantitatively as well as determine product distributions bulk electrolysis experiments were conducted.

### 3.2 Product distributions and FEs of vanillin bulk electrolysis experiments

**Fig. 4:**
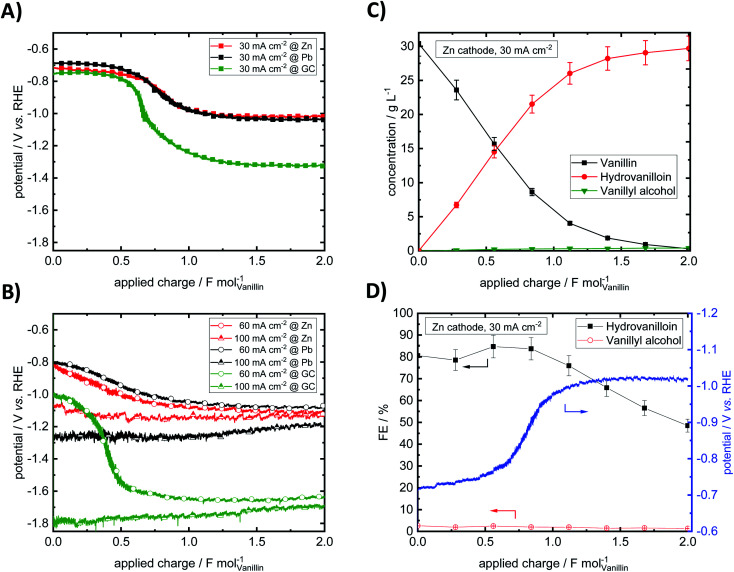
Potential–charge plots for vanillin reduction at Pb, Zn and GC at a current density of 30 mA cm^−2^ (A) and at current densities of 60 and 100 mA cm^−2^ (B). Exemplary concentration course of the electrochemical vanillin reduction. Analysis of the catholyte was carried out by CE-UV (C). Calculated FEs for hydrovanilloin and vanillyl alcohol (D). Parameters: vanillin concentration 0.2 M in 1 M sodium hydroxide solution, catholyte stirred at 250 rpm, RT.

Bulk electrolysis of vanillin in 1 M NaOH was performed at three different current densities 30, 60 and 100 mA cm^−2^ at a fixed applied charge of 2 F mol^−1^. Potential–charge plots in galvanostatic electrolysis mode at Pb, Zn and GC cathodes are shown in Fig. 4A and B. Product yields, mass differences of cathodes in the electrolysis and potentials at start and end of electrolysis are summarized in Table 1.

**Table 1:** Product yields, mass differences of cathodes and potentials at the start and end of electrolysis of the electrochemical vanillin reduction after an applied charge of 2 F mol_Vanillin_^−1^^a^

Cathode material	Current density (mA cm^−2^)	Mass difference cathode (mg)	Vanillin conversion (%)	Yield hydrovanilloin (%)	Yield vanillyl alcohol (%)	Potential at start (mV *vs.* RHE)	Potential at end (mV *vs.* RHE)
Zn	30	+0.21	99.0	97.7	1.3	−720	−1018
	60	+0.00	90.7	88.9	1.8	−824	−1112
	100	−0.09	80.1	78.6	1.5	−1073	−1134
Pb	30	−2.86	99.9	99.1	0.9	−686	−1040
	60	−5.90	95.0	93.4	1.6	−803	−1083
	100	−19.30	93.7	87.1	6.6	−1266	−1195
GC	30	+0.05	97.9	95.9	2.0	−750	−1326
	60	+0.12	92.9	78.6	14.3	−1012	−1640
	100	+0.13	88.0	74.9	13.1	−1791	−1696

a Electrolysis was performed under following conditions – catholyte: 0.2 M vanillin in 1 M sodium hydroxide, anolyte: 1 M sodium hydroxide, room temperature, catholyte stirred at 250 rpm, potentials were corrected by electrolyte resistance measured by GEIS. Catholyte was analyzed by CE-UV.

Throughout the electrolysis at 30 and 60 mA cm^−2^ the cathode potential drops to more negative values due to decreasing vanillin concentration and increasing conversion resulting in higher overpotentials for the electrochemical reduction of vanillin. For example, at 30 mA cm^−2^ at an applied charge around 0.60 F mol^−1^ for GC and 0.75 F mol^−1^ for Pb and Zn the potential shifts to a new more negative plateau, where preferably the HER is taking place. At this stage the vanillin electrolysis is under mass transfer control. An exemplary progression of the electrochemical vanillin reduction for a Zn cathode at 30 mA cm^−2^ is shown Fig. 4C and D. A change of the slope of the vanillin conversion is observed transitioning from a linear (kinetically controlled) to an exponential (mass transport controlled) concentration decrease. At 100 mA cm^−2^ no drop of the cathode's potential is observed suggesting that the reaction is under full mass transport control from start on and fastest conversion rates are achieved under given conditions. Potentials at the start and at the end of the electrolysis decrease with increasing current density due to a stronger cathodic polarization of the electrodes. Similar current–potential–charge behavior was observed by Jow *et al.* at a mercury pool electrode.^18^ For example, starting potentials for a 0.033 M vanillin solution in 1 M NaOH decreased from −1.72 V *vs.* SCE at 30 mA cm^−2^ to −2.05 V *vs.* SCE at 100 mA cm^−2^. Potential shifts to the HER plateau were slightly earlier at applied charges of 0.1–0.5 F mol^−1^, which may be due to slower mixing of the catholyte and different cell and electrode design. Here, for 30 and 60 mA cm^−2^ the cathode's potentials for the three materials Zn, GC and Pb at the start of the electrolysis are approximately similar and are in the range between −0.68 and −0.824 V *vs.* RHE except for GC at 60 mA cm^−2^, where a potential at the start of −1.01 V *vs.* RHE is observed. At the highest current density of 100 mA cm^−2^ and in the mass transport limited region of the electrolysis the potential is dominated by the HER. Due to high HER overpotentials very negative potentials up to −1.79 V *vs.* RHE are reached either at GC at 30 and 60 mA cm^−2^ in the mass transport limited regions or at 100 mA cm^−2^ at Pb and GC throughout the whole electrolysis. Minor HER overpotentials at Zn compared to the other two investigated cathode materials hinder the potential from reaching very negative values being at maximum −1.13 V *vs.* RHE at 100 mA cm^−2^.

Almost no mass difference for the Zn and GC electrodes are found after electrolysis for all investigated current densities deducing stable behavior. Mass differences range from −0.09 to + 0.21 mg and + 0.05 to + 0.13 mg for Zn and GC, respectively. Small positive changes of the cathode's mass may be due to small deposits of vanillin or reaction products on the electrode surface and measurement errors. However, for reproducible potential courses at GC intensive polishing of the electrode surface is necessary showing some change of the morphology under given conditions. This agrees well with observations by Chandrasekaran *et al.*, who found a sensitive behavior to the pre-treatment by means of polishing of GC cathodes for the reduction of vanillin in aprotic and aqueous media.^42^ For Pb significant loss of electrode material was observed. Mass differences were in the range between −2.86 to −19.30 mg per electrolysis run. Similar cathodic corrosion of lead was observed *e.g.* by Gütz *et al.*, who used leaded bronzes as alternative cathode material for electro-reduction such as deoxygenation, dehalogenations and hydrodimerization.^36^

Nearly full conversion of vanillin was achieved after an applied charge of 2 F mol^−1^ at all three electrode materials at 30 mA cm^−2^. Hydrovanilloin is the main product in all cases and vanillyl alcohol formation is neglectable with a maximum yield of 2% at GC. With increasing current density vanillin conversion is decreasing at the fixed applied charge of 2 F mol^−1^, because the current is consumed by the competing HER. At 100 mA cm^−2^ the lowest vanillin conversion of 80.1% is observed for Zn. At 100 mA cm^−2^, however, the highest selectivity for the pinacolization product hydrovanilloin is achieved for Zn among the three investigated cathode materials with neglectable yields of vanillyl alcohol of 1.5%. In comparison significant vanillyl alcohol yields of 13–14% are observed for GC at 60 and 100 mA cm^−2^ as well as 6.6% for Pb at 100 mA cm^−2^.

**Fig. 5:**
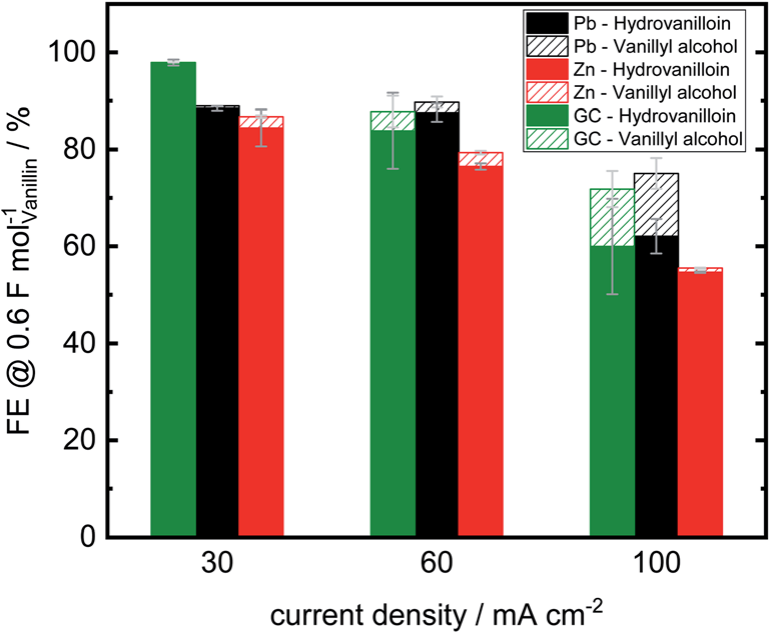
Faradaic efficiencies for hydrovanilloin and vanillyl alcohol formation in electrochemical bulk electrolysis of vanillin after 0.6 F mol^−1^ (vanillin concentration 0.2 M in 1 M sodium hydroxide solution, catholyte stirred at 250 rpm, RT). Error bars indicate highest and lowest values of two electrolysis runs.

Analogously to Jow *et al.* FEs of the reduction products hydrovanilloin and vanillyl alcohol after an applied charge of 0.6 F mol^−1^ were calculated.^18^ At this stage the reaction is still kinetically controlled at low current densities and FEs stay constant (see Fig. 4D).^43^ For proving reproducibility, the experiments for the FE evaluation were conducted two times and the resulting mean values are presented in Fig. 5. At the lowest current density of 30 mA cm^−2^ FEs for hydrovanilloin formation are in the range of 85–88% for Pb and Zn, whereas for GC a FE of ≈98% is obtained due to its higher overpotential for the competing HER. FEs for vanillyl alcohol are below 0.5% for Pb and GC. A slightly higher FE for vanillyl alcohol formation at Zn cathodes of 2.3% is observed. Comparatively, no vanillyl alcohol formation was reported by Jow *et al.* for a Hg cathode in the current density range of 15–100 mA cm^−2^. FEs for hydrovanilloin decreased from 80% to 45% when increasing the current density from 15 mA cm^−2^ to 100 mA cm^−2^. But it should be noted that the starting vanillin concentration was lower being 33 mM of vanillin in 1 M NaOH.^18^ Here, total FEs of vanillin reduction products decrease gradually to 71–75% at 100 mA cm^−2^ for Pb or GC and to 55% for Zn. However, comparable FEs for hydrovanilloin formation of 55–62% are achieved among the three investigated cathode materials, since the FE for vanillyl alcohol formation stays at low percentages for Zn, whereas FEs of 12–13% for vanillyl alcohol are obtained for GC and Pb.

According to the published literature the dissociated hydroxyl group in basic aqueous media leads to a decrease of the stability of the metal ketyl intermediate due to electron release to the benzene ring and therefore to a preferably dimerization reaction pathway (see Scheme 1). Significant vanillyl alcohol formation is only observed at very negative potentials, where kinetics for the second electron transfer to the metal ketyl intermediate would be fast enough compared to the dimerization pathway.^18^ These negative potentials are either reached herein at Pb and GC electrodes at higher current densities or in mass transport controlled region of the electrolysis. For example, significant vanillyl alcohol formation at GC at 60 mA cm^−2^ is only observed after an applied charge of 0.5 F mol^−1^, when the potential drops from −1 V *vs.* RHE to the HER plateau of ≈−1.6 V *vs.* RHE (ESI, Fig. S3†). Therefore, FEs at 0.6 F mol^−1^ for GC at 60 mA cm^−2^ are still low, although a high vanillyl alcohol yields is observed after an applied charge of 2 F mol^−1^. It is assumed that the comparable lower HER overpotential depolarizes the Zn electrode hindering the potential of reaching very negative values and therefore inhibiting the second electron being transferred to the metal ketyl intermediate. As a result, the highest selectivity for hydrovanilloin at higher current densities is obtained for Zn and side product formation of vanillyl alcohol is neglectable. The negative impact of the minor HER overpotential at Zn on the FE of hydrovanilloin is low showing comparable FEs of ≈55% to Pb and GC up to high current densities. Furthermore, it is interestingly to note that the viable potential window of Zn for electroreductions in aqueous alkaline media is very limited. At potentials higher and equal to the open circuit potential of −0.450 V *vs.* RHE corrosion occurs and at potentials more negative than −0.800 V *vs.* RHE a significant increase of the competing HER is observed. The electroreduction of vanillin with an onset potential of −0.575 V *vs.* RHE at Zn seems to fit perfectly in the viable potential window.

### 3.3 Product isolation and identification of the electrochemical vanillin reduction

**Fig. 6:**
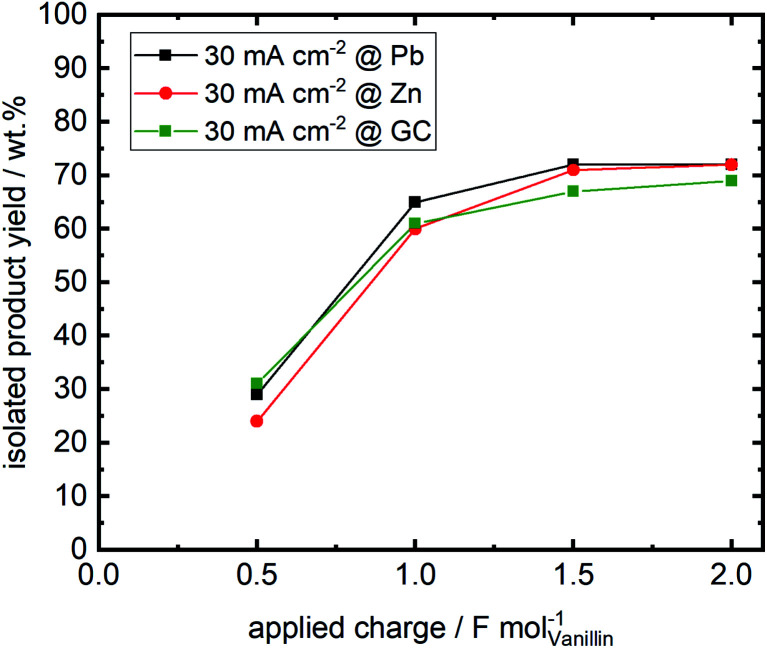
Isolated product yields *versus* applied charge for the three investigated cathode materials Zn, Pb and GC. Products were isolated by titrating the catholyte to pH = 2 with 1 M HCl followed by filtrating, washing and drying.

Isolated product yields obtained by precipitation after acidification of the catholyte differ from the analyzed hydrovanilloin amount in the catholyte solution. Fig. 6 shows the isolated product yields at 30 mA cm^−2^ for the three investigated cathode materials *versus* the applied charge. Isolated yields at 60 and 100 mA cm^−2^ at an applied charge of 2 F mol^−1^ are show in the (ESI, Table S1†). A maximum isolated yield of 72% is achieved after an applied charge of 2 F mol^−1^ for Zn and Pb cathodes at 30 mA cm^−2^, although almost full vanillin conversion to hydrovanilloin is observed in the directly analyzed catholyte (see Table 1). Similar isolated yields are reported in the literature ranging between 69 and 76% for applied charges of 1.1 F mol^−1^ and 1.56 F mol^−1^, respectively.^17,44^ Published yields of 86% by Amarasekara *et al.* could not be reproduced, even though isolation was performed similar titrating the catholyte to pH = 2 with HCl and having almost full vanillin conversion. Neither current densities nor applied charges were mentioned.^23^ Maximum isolated yields of 69% for the electroreduction of vanillin at GC are slightly lower compared to Pb and Zn due to slightly higher formation of vanillyl alcohol, which remains in the filtrate. The isolated product yield generally decreases with increasing current density from 30 to 100 mA cm^−2^ at a fixed applied charge of 2 F mol^−1^ due to the increasing charge consumption of the competing HER. An isolated yield of 49% is reported for the Zn cathode at 100 mA cm^−2^. The isolated product was identified by HPLC-DAD-MSD, FT-IR and ^1^H-, ^13^C- and 2D-NMR (HSQC, ^13^C/^1^H) as pure hydrovanilloin (ESI, Fig. S4–S6†), which agrees with the published literature.^17,23^ This shows that the product isolation by the commonly used titration step is sub optimal giving space for further improvement, *e.g.* by membrane technology. Interestingly to note is that the pinacolization at Zn electrode exhibits the same stereoselectivity as it was previous reported for Pb electrode. The pinacolization can lead two different diastereomeric forms of hydrovanilloin, namely the dl-form and the meso-form. Their ratio can be determined from the ^1^H-NMR methoxy group signals or the Cα–Hα/Cα′–Hα′ signals in the aliphatic region of hydrovanilloin. Here, a meso- to dl-form ratio of 78% is calculated, which agrees well with the literature showing a ratio of ≈80% at 30 mA cm^−2^ synthesized at a Pb cathode.^21^ However, it should be mentioned that the meso- to dl-form ratio is dependent on the applied current density and *e.g.* a maximum ratio of 94% was achieved by Amarasekara *et al.* at 64 mA cm^−2^ at Pb cathodes.^21^

### 3.4 Linear sweep voltammetry of divanillin

**Fig. 7:**
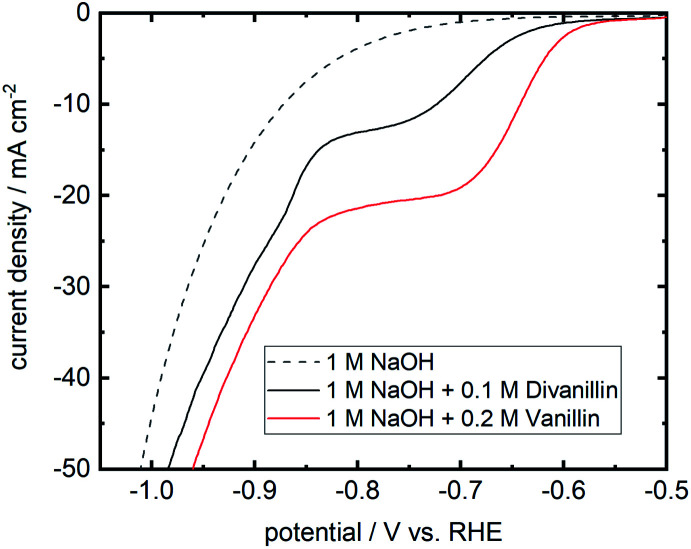
Cathodic LSV for divanillin reduction in alkaline aqueous media at a Zn cathode in static solution. Potential sweep rate is 10 mV s^−1^. Background current in pure electrolyte is shown as dashed line. For comparability the LSV of a 0.2 M vanillin aqueous alkaline solution at a Zn cathode is shown in red.

The molecular weight build-up of divanillin to polyvanillin is carried out by electroreduction of the two aldehyde groups of divanillin leading to the pinacol coupling product. Fig. 7 shows the LSV of divanillin in aqueous alkaline solution at a Zn cathode and for comparability the LSV of vanillin. It was expected that for the same number of aldehyde groups of vanillin and divanillin onset potentials and reduction currents would be equal due to their similar molecular structure and the far distance between the aldehyde groups of divanillin. For example, the electrochemical reduction behavior of isophthalaldehyde bearing two aldehyde groups is similar to the electroreduction of benzaldehyde in acidic and alkaline media at twice the concentration.^45^ However, the electroreduction product of benzaldehyde in acidic and alkaline media is the corresponding alcohol.^46^ For the electroreduction of vanillin the main product in alkaline media is the pinacol product hydrovanilloin due to the induced instability of the dissociated phenolic group in *para*-position of the aldehyde leading preferably to dimerization.^18^ The onset potential of divanillin is shifted 50 mV in negative direction and cathodic currents are inferior compared to the vanillin reduction at twice the concentration. It is therefore assumed that the electroreduction of one aldehyde group of divanillin is affecting the electroreduction of the second aldehyde group. The resulting negative charge on the carboxyl group due to the electron transfer may be delocalized by the aromatic rings leading to a more difficult reduction of the second aldehyde group. Moreover, the two phenolic groups of divanillin are dissociated in the aqueous alkaline electrolyte increasing the total negative charge of the molecule. Indication of a second electroreduction peak from the second aldehyde can just be suspected at a potential of ≈−850 mV *vs.* RHE, since at these negative potentials reduction currents of the competing HER strongly overlap. As for the electroreduction of vanillin current densities up to 100 mA cm^−2^ were achievable, maximum applied currents for the divanillin reduction have to be reduced in the following bulk electrolysis experiments. A maximum applied current density of 60 mA cm^−2^ is chosen.

### 3.5 Bulk electrolysis of divanillin to polyvanillin

**Fig. 8:**
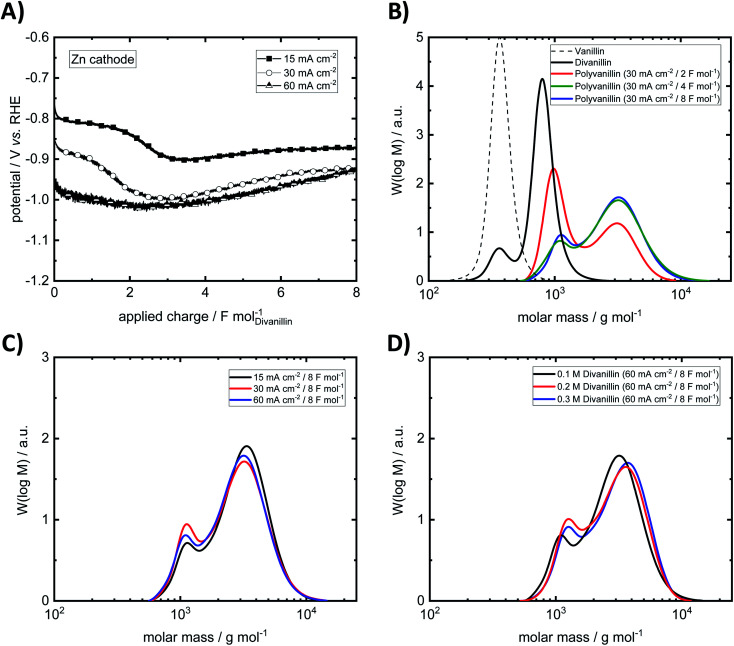
(A) Potential–charge plots of divanillin reduction. (B) Exemplary molecular weight build-up from vanillin to divanillin followed by cathodic polymerization to polyvanillin at 30 mA cm^−2^ up to an applied charge of 8 F mol^−1^. (C) Impact of current density on the molecular weight and shape of polyvanillin. (D) Impact of concentration on the molecular weight and shape of polyvanillin (0.2 M and 0.3 M divanillin reduction was performed with second batch of divanillin). Electrolysis was performed under following conditions – cathode: Zn (5 cm^2^), anode: Ni-foam, catholyte: 0.1 M divanillin in 1 M sodium hydroxide (carried out with first batch of divanillin containing 4.9 wt% vanillin, if not stated otherwise), anolyte: 1 M sodium hydroxide, room temperature, catholyte stirred at 250 rpm, isolated products were analyzed by SEC. SEC was calibrated *versus* pullulan standard.

Since in the vanillin reduction experiments Zn was found as excellent replacement for Pb as cathode material for hydrodimerization, bulk electrolysis experiments of divanillin are primarily conducted with Zn as cathode. Bulk electrolysis experiments of divanillin at the Zn cathode are summarized in Table 2 and Fig. 8A–D.

**Table 2:** Impact of current density, applied charge and divanillin concentration on isolated yields and molecular weights of isolated polyvanillin^a^

Run	Current density (mA cm^−2^)	Applied charge (F mol^−1^)	Divanillin concentration (mol L^−1^)	Isolated yield (%)	*M*_W_ b (g mol^−1^)	*M*_N_ b (g mol^−1^)	*M*_W_/*M*_N_
1	15	2	0.1	57.9	1771	1360	1.30
2	15	4	0.1	66.2	3132	2321	1.35
3	15	8	0.1	64.1	3171	2362	1.34
4	30	2	0.1	74.6	2095	1464	1.43
5	30	4	0.1	64.2	3103	2174	1.42
6	30	8	0.1	69.9	3023	2186	1.38
7	60	2	0.1	71.2	1687	1239	1.36
8	60	4	0.1	64.8	2864	1748	1.64
9	60	8	0.1	69.7	3016	2201	1.37
10	60	8	0.2^c^	72.8	3082	2260	1.36
11	60	8	0.3^c^	81.1	3208	2344	1.37

a Electrolysis was performed under following conditions – cathode: Zn (5 cm^2^), anode: Ni-foam, catholyte: 0.1 M divanillin in 1 M sodium hydroxide (first batch of divanillin containing 4.9 wt% vanillin, if not stated otherwise), anolyte: 1 M sodium hydroxide, room temperature, catholyte stirred at 250 rpm, isolated products were analyzed by SEC.

b Calibrated *versus* pullulan standard.

c Second batch of divanillin containing no vanillin.

At the lowest applied current density of 15 mA cm^−2^ the reaction is kinetically controlled showing a drop of the cathode potential from −800 mV *vs.* RHE to −900 mV *vs.* RHE at an applied charge of ≈2 F mol^−1^ due to decreasing divanillin concentration (Fig. 8A). In the latter part of the reaction the divanillin reduction is under mass transport control and, as in the electrochemical vanillin reduction, the competing HER is the dominating reaction. No potential drop at a current density of 60 mA cm^−2^ corresponding to a cathode potential of ≈−1 V *vs.* RHE is observed indicating that the reaction is under mass transport control respectively highest possible conversion rate from start on. The slight increase of the cathodic potential with increasing applied charge in the mass transport-controlled region is explained by the decreasing resistance of the electrolyte in the cathodic chamber, which was typically 0.5 Ω at an applied charge of 8 F mol^−1^. On the basis of charge neutrality Na^+^-ions migrate through the ion selective membrane from the anodic to the cathodic chamber leading to a lower resistance between the working and the reference electrode on the cathode side and therefore to a lower recorded potential with increasing reaction time.

An exemplary molecular weight build-up from vanillin to polyvanillin at the Zn cathode for a current density of 30 mA cm^−2^ in the reductive polymerization step of divanillin is shown in Fig. 8B. Since in the first batch of the enzymatic conversion of vanillin to divanillin a remaining vanillin fraction of 4.9 wt% was found, a small pre-peak in the SEC spectra of divanillin is observed. It should be noted that vanillin and divanillin are no polymeric compounds and have an exactly weight of 152 g mol^−1^ for vanillin and 302 g mol^−1^ for divanillin. Due to their low molecular weight such substances are usually not appropriate for SEC analysis and the broad molecular weight distributions (MWDs) do not correspond to their actual molecular weight. However, these compounds are shown in Fig. 8B for a better visualization of the process. The actual polymerization of divanillin to polyvanillin shows a bimodal frequency distribution of molar mass under given electrochemical process conditions. A first local maximum is observed at a molecular weight of ≈1000 g mol^−1^ and a second local maximum at ≈3200 g mol^−1^. With increasing reaction time or applied charge the first local maximum decreases and the second local maximum increases until a final state of the MWD shape is reached. The first local maximum becomes more and more a shoulder. This result is observed for all three investigated current densities of 15, 30 and 60 mA cm^−2^ (ESI, Fig. S7 and S8†). Similar bimodal molecular weight distributions were *e.g.* observed for the emulsion polymerization of ethylene^47^ or isotactic polystyrene.^48^ No further change of the shape of the MWD is observed after an applied charge of 4 F mol^−1^ (double theoretical charge for full divanillin pinacolization) for 15 and 30 mA cm^−2^, whereas this shape is achieved after 8 F mol^−1^ (quadruple theoretical charge for full divanillin pinacolization) at the highest current density of 60 mA cm^−2^. The most energy efficient polymerization is therefore conducted at low current densities, where the competing HER is still slow, whereas fastest conversion is conducted at 60 mA cm^−2^ under mass transport control for the given conditions. The formation of the bimodal frequency distribution of molar mass was at first suggested due to remaining vanillin in the divanillin, since vanillin showed a higher activity compared to divanillin. This would lead to fast formation of small pre-polymers followed by the actually main polymerization of divanillin. However, polyvanillin synthesis experiments with a second divanillin batch containing no remaining vanillin resulted in a similar shape and similar polymerization degrees (see Fig. 8D, run 10–11). Therefore, the influence of the remaining vanillin fraction of 4.9 wt% in the first batch can be neglected and the bimodal frequency distribution of molar mass still remains subject of further studies.

Further it is found that the maximum degree of polymerization is independent of the applied current density at a Zn cathode up to potentials of ≈−1 V *vs.* RHE, which corresponds to a current density of 60 mA cm^−2^ (Fig. 8C). Weight average molecular weights *M*_W_ up to ≈3200 g mol^−1^ are achieved. The polydispersity *M*_W_/*M*_N_ is relatively low showing a narrow distribution. Values for the polydispersity lie in the range between 1.30 and 1.43 excluding run 8, where a polydispersity of 1.64 is determined. This higher value, however, is calculated for a partly polymerized sample at a current density of 60 mA cm^−2^ and an applied charge of 4 F mol^−1^ showing almost two equally high peaks in the bimodal frequency distribution of molar mass (ESI, Fig. S8†).

Since higher concentrations of divanillin might lead to an increase of the polymerization degree, polyvanillin synthesis was performed for higher concentration of divanillin up to 0.3 M (run 10–11). A higher divanillin concentration would lead to an increased radical intermediate concentration at the electrode's surface, thus, might increase pinacol formation (radical combination) instead of alcohol formation terminating polymer chain growth. Similar observation were shown for the electroreduction of vanillin to hydrovanilloin.^18^ However, no increase of the polymerization degree was found when tripling the concentration from 0.1 M to 0.3 M divanillin (Fig. 8D).

Isolated yields ranged between 58% and 74% at a divanillin concentration of 0.1 M showing no discernible trend when varying the current density and the applied charge. The results suggest that reaction products are in solubility equilibrium when titrating the alkaline catholyte to pH = 2 for product precipitation also showing potential for improvement in the isolation step as in the hydrovanilloin synthesis. However, an increase of the isolated yields is observed for higher concentrations reaching values of 81% at a divanillin concentration of 0.3 M. Isolated polyvanillin yields by Amarasekara *et al.* for a 0.175 M divanillin solution were slightly higher being 91% after an applied charge of 35.2 F mol^−1^.^23^

Finally, it is interestingly to note, that the reductive pinacolization forms soluble polymers. Since on the other hand, the oxidative polymerization for example of pyrrole, aniline or thiophene lead to the formation of electronic conducting polymer films on the anode.^49,50^ No deposition of polymers on Zn cathodes was observed throughout all polyvanillin electrolysis experiments.

As Pb cathodes were used in the original synthesis of polyvanillin by Amarasekara *et al.*^23^ and GC cathodes also showed reasonable activity in the electrochemical vanillin reduction experiments, the impact of different cathode materials were further investigated. The applied charge was selected to be 8 F mol^−1^, since full polymerization was achieved at Zn cathodes at this amount of applied charge. The molecular weight data of the polyvanillin samples synthesized at GC and Pb cathodes are summarized in Table 3. Potential–charge plots and MWDs are shown in the (ESI, Fig. S9–S11†).

**Table 3:** Molecular weight data of synthesized polyvanillin at Pb and GC cathodes^a^

Cathode material	Current density (mA cm^−2^)	Applied charge (F mol^−1^)	*M*_W_ b (g mol^−1^)	*M*_N_ b (g mol^−1^)	*M*_W_/*M*_N_
Pb	15	8	2444	2023	1.21
Pb	30	8	2958	2285	1.29
Pb	60	8	1898	1501	1.26
GC	15	8	2986	2384	1.25
GC	30	8	3382	2631	1.29
GC	60	8	1264	1017	1.24

a Electrolysis was performed under following conditions – anode: Ni-foam, catholyte: 0.1 M divanillin in 1 M sodium hydroxide, anolyte: 1 M sodium hydroxide, room temperature, catholyte stirred at 250 rpm, isolated products were analyzed by SEC.

b Calibrated *versus* pullulan standard.

Bimodal frequency distributions of molar mass are also found for GC and Pb cathodes in all cases. Weight-average molecular weights *M*_W_ of ≈3000 g mol^−1^ are approximately similar to polyvanillin synthesized at Zn cathodes for the current densities of 15 and 30 mA cm^−2^. A significant drop of the weight-average molecular weight is observed at Pb and GC at the highest applied current density of 60 mA cm^−2^ to 1898 g mol^−1^ and 1264 g mol^−1^, respectively. A high amount of a low molecular weight fraction still exists in the polyvanillin sample synthesized at the GC cathode showing a high first local maximum in the bimodal frequency distributions of molar mass at ≈800 g mol^−1^. Low polydispersities *M*_W_/*M*_N_ ranging between 1.21 and 1.29 are found for synthesized polyvanillin at GC and Pb cathodes also showing very narrow MWDs. Cathode potentials are significant more negative compared to Zn reaching values of −1.30 V *vs.* RHE and −1.75 V *vs.* RHE for Pb and GC at a current density of 60 mA cm^−2^, respectively. This agrees well with obtained results in the electrochemical vanillin reduction experiments. A decrease of the cathode's potential is observed at 60 mA cm^−2^ for Pb and GC at the start of the electrolysis indicating that the reaction is not under full mass transport control from begin on, which is due to higher overpotentials of the competing HER.

### 3.6 NMR analyses of polyvanillin

**Fig. 9:**
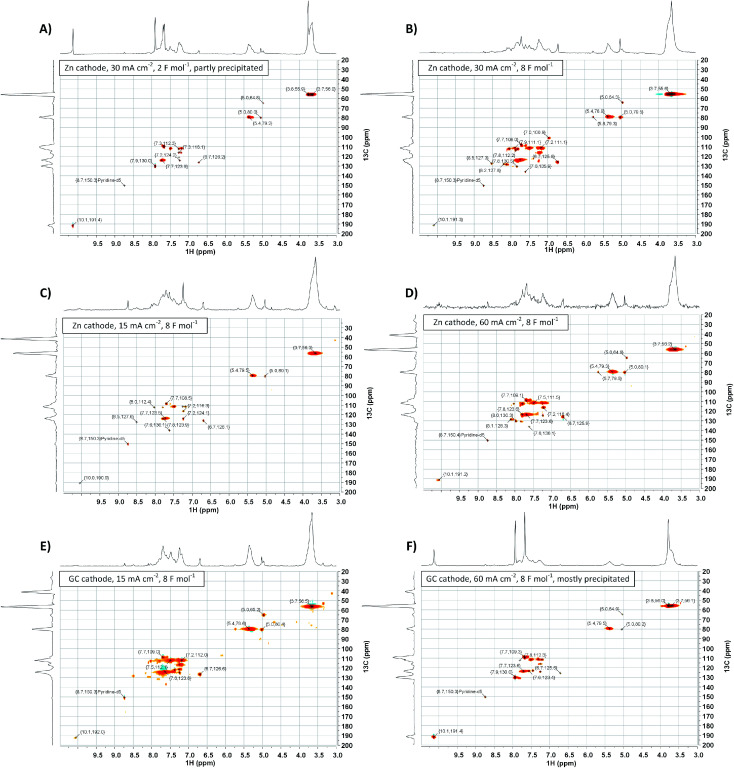
2D-NMR (HSQC, ^13^C/^1^H) spectra of selected polyvanillin samples: (A) Zn cathode, 30 mA cm^−2^, 2 F mol^−1^ (partly precipitated). (B) Zn cathode, 30 mA cm^−2^, 8 F mol^−1^. (C) Zn cathode, 15 mA cm^−2^, 8 F mol^−1^. (D) Zn cathode, 60 mA cm^−2^, 8 F mol^−1^. (E) GC cathode, 15 mA cm^−2^, 8 F mol^−1^. (F) GC cathode, 60 mA cm^−2^, 8 F mol^−1^ (mostly precipitated).

**Fig. 10:**
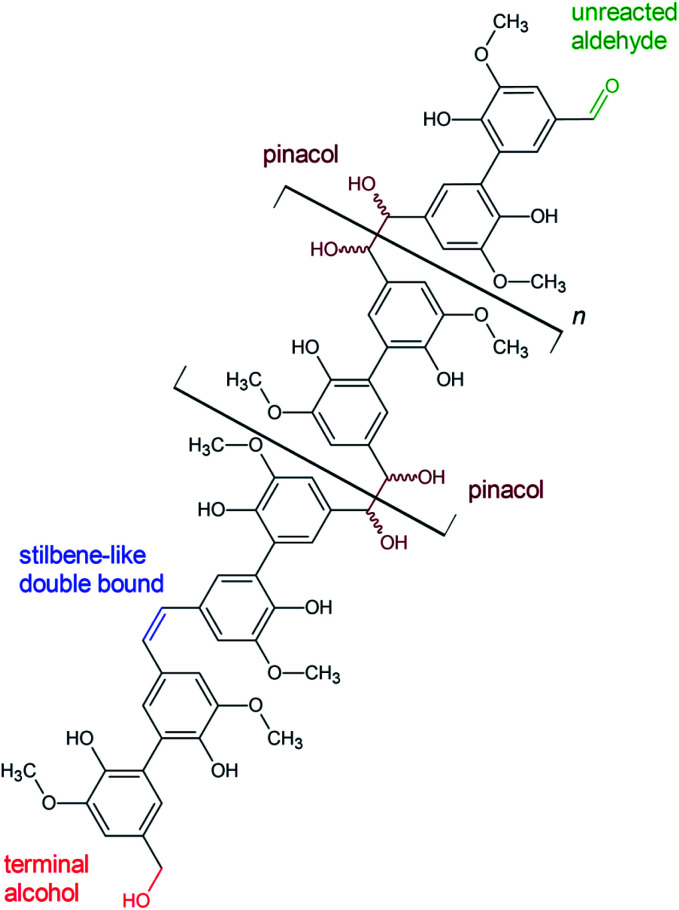
Proposed structural features of polyvanillin based on the results of 2D-NMR (HSQC) analysis.

To gather more insight in structural features of the synthesized polyvanillin 2D-NMR (HSQC, ^13^C/^1^H) spectra of selected sampled were recorded combining the sensitivity of ^1^H-NMR with the higher resolution of ^13^C-NMR (Fig. 9, 10 and Table 4).^51^ The polyvanillin samples were fully soluble in pyridine-d_5_ except for the sample synthesized at a Zn cathode at a current density of 30 mA cm^−2^ and a low applied charge of 2 F mol^−1^ (Fig. 9A), which partly precipitated and for the sample synthesized at a GC cathode at a current density of 60 mA cm^−2^ and an applied charge of 8 F mol^−1^ (Fig. 9F), which mostly precipitated. In both precipitated cases low molecular weights were observed in the SEC analysis.

**Table 4:** Identified cross-coupling signals in the 2D-NMR (HSQC, ^13^C/^1^H) spectra of polyvanillin samples, their assessment and some additional notes

Cross-coupling signals *δ*^13^C/*δ*^1^H in ppm	Assessment	Note
≈55.60/3.7	(CH_3_–O-Ar)	Methoxyl group
64.9/5.0	R–CH_2_-OH	Linked to primary aliphatic OH-group, sometimes weak signal
79.5/5.0 & 78.9/5.4	Cα′–Hα′, Cα′′–Hα′′	Vicinal OH groups in aliphatic region
109.0/7.7	C2–H2	C5-substituted guaiacyl, cross-coupling *δ*^13^C/*δ*^1^H aromatic ring
123.6/7.7	C6′–H6′	Cross-coupling *δ*^13^C/*δ*^1^H aromatic ring
126.6–125.6/6.7	Cα′–Hα′ & Cα′′–Hα′′	Stilbene like double bond instead of vicinal OH groups
130.0/7.9	C6–H6	C5-substituted guaiacyl, cross-coupling *δ*^13^C/*δ*^1^H aromatic ring
191.4/10.1	Cα–Hα	Aldehyde end group, sometimes weak signal
123.87/7.220; 135.91/7.580 & 150.35/8.740	C–H cross-coupling in pyridine	Pyridine-d_5_, 99%ical
111.5/7.5; 116.4/7.2; 121.5/7.3	Unknown	Could be cross-coupling signals of ^13^C/^1^H in aromatic rings, differing in chemical environment

The existents of structural features of polyvanillin were confirmed in all cases. Cross-coupling signals of the methoxy groups coupled to the aromatic rings at *δ*_H_ = 3.7 ppm/*δ*_C_ = 55.60 ppm were found in every sample. The vicinal hydroxy groups in the aliphatic region generated by the pinacol formation (see Fig. 10, brown motif) were assigned to the cross-coupling signals at *δ*_H_ = 5.0 ppm/*δ*_C_ = 79.5 ppm and *δ*_H_ = 5.4 ppm/*δ*_C_ = 78.9 ppm and cross-coupling signals from the aromatic ring of the C_5_-substituted guaiacol to *δ*_H_ = 7.7 ppm/*δ*_C_ = 109.0 ppm, *δ*_H_ = 7.7 ppm/*δ*_C_ = 123.6 ppm and *δ*_H_ = 7.9 ppm/*δ*_C_ = 130.0 ppm (Table 4). Very broad peaks of the methoxy groups signals in the ^1^H-spectra confirm the high molecular weight of the polyvanillin samples (Fig. 9B–E). In the two samples with lower molecular weight (Fig. 9A and F) ^1^H-signals from the methoxy group are split into a high sharp peak and a lower broader peak verifying the existence of a very low molecular fraction. Moreover, very sharp peaks are also observed in the aromatic region in the ^1^H-spectra between 6.5 ppm and 8.5 ppm further confirming the existence of a very low molecular fraction. A cross-coupling signal observed at *δ*_H_ = 10.1 ppm/*δ*_C_ = 191.4 ppm was assigned to the unreacted aldehyde end groups (see Fig. 10, green motif). Only very weak to none signals at all from the remaining unreacted aldehyde end groups can be seen in the spectra of the high molecular weight samples (Fig. 9B–E) confirming full conversion divanillin for the Zn cathode after an applied charge of 8 F mol^−1^ at the three current densities 15, 30 and 60 mA cm^−2^ and for the GC cathode at the current density of 15 mA cm^−2^. As expected a strong signal of unreacted aldehyde groups is found in the ^1^H-NMR spectra for the party polymerized sample at the Zn cathode at 30 mA cm^−2^ when only applying a charge of 2 F mol^−1^ (Fig. 9A). Surprisingly, unreacted aldehyde groups are also found in the polyvanillin sample synthesized at the GC cathode at 60 mA cm^−2^ after an applied charge of 8 F mol^−1^ (Fig. 9F). This explains the low molecular weight analyzed in the SEC analysis. Since a drop of the cathode's potential at the start of the electrolysis was observed indicating some conversion of divanillin, it is assumed that the main reaction in the latter period of the reaction is the HER leading to no further divanillin reduction. At the moment this phenomenon cannot be explained and is subject of further studies. Besides undergoing pinacolization the aldehyde end groups can be reduced by a 2e^−^ reduction to the corresponding alcohol terminating the polymer chain growth (see Fig. 10, red motif). As shown in the electrochemical vanillin reduction experiments and by Jow *et al.* the ratio between alcohol formation and pinacolization increases with decreasing potential respectively under more severe conditions in favor of alcohol formation.^18^ The cross-coupling signal of the primary, terminal alcohol group was assigned to the peak at *δ*_H_ = 5.0 ppm/*δ*_C_ ≈ 64.9 ppm. For the polyvanillin samples synthesized at Zn cathodes the amount of terminal alcohol groups slightly increases with increasing current density respectively decreasing cathode potential and increasing applied charge (Fig. 9A–D). At GC comparably, a higher amount of these terminal alcohol groups is already observed at 15 mA cm^−2^ due to more negative potentials. In the sample synthesized at a current density of 60 mA cm^−2^ the absolute peak height of the terminal alcohol groups is lower than at a current density of 15 mA cm^−2^. However, in this partly polymerized sample the ^1^H-NMR signal of the primary, terminal alcohol group is still relatively high compared to the ^1^H-NMR signal of the pinacol groups. Moreover, an increasing signal at *δ*_H_ = 6.7 ppm/*δ*_C_ ≈ 126.0 ppm is observed with decreasing cathode potential respectively under more severe conditions. This signal was assigned to stilbene-like double bond systems, since the signal is located in the unsaturated and aliphatic region of the ^1^H/^13^C-NMR spectra (see Fig. 10, blue motif). Unfortunately, it cannot be distinguished whether these stilbene-like double bond systems are created during the isolation process by titrating with concentrated HCl or in the actual electrolysis. However, a clear trend of increasing double bond systems with harsher electrolysis conditions is observed favoring the case of stilbene formation in the electrolysis. It is suspected that the generated vicinal alcohol groups in the aliphatic region generated by pinacolization are converted to these stilbene-like double bond systems by elimination of water followed by a further reduction to the corresponding double bond. Similar hetero-catalyzed reductions have been shown for the conversion of acetylated hydrovanilloin, which is the pinacol product of vanillin, to the stilbene and stilbene epoxides to alkenes.^44,52,53^

Furthermore, ^31^P-NMR analyses of selected polyvanillin samples were conducted for quantification of phenolic and aliphatic OH groups. In theory, the polyvanillin molecule should be characterized by having more or less the same molar number of phenolic and aliphatic OH groups. By means of ^31^P-NMR analysis, after *in situ* labeling Cl-TMDP, this should be illustratable. An exemplary ^31^P-NMR spectrum is shown in the (ESI, Fig. S12†) and quantitative calculated results are summarized in Table 5. Contrary to theoretical considerations, the polyvanillin samples prepared in this study often exhibit a molar ratio of aliphatic to phenolic OH groups of approximately 0.5. This finding can of course be discussed speculatively with respect to a steric hindrance of the *in situ* labeling process or an uncontrolled water separation during the isolation from the electrolyte. As no conclusive answer could be given for this yet, further studies will get to the bottom of this preliminary result. However, the low observed degree of polymerization in the SEC analysis for the polyvanillin sample synthesized at GC at a current density of 60 mA cm^−2^ is confirmed by the ^31^P-NMR results showing almost no generated aliphatic hydroxy groups by pinacolization.

**Table 5:** Selected results of ^31^P-NMR-analyses on different polyvanillin samples, after *in situ* labelling with 2-chloro-4,4,5,5-tetramethyl-1,3,2-dioxaphospholane (Cl-TMDP) in solvent pyridine/CDCl_3_ = 1.6/1 (v/v), ISTD: cyclohexanol, Bruker Avance 300 MHz

Cathode material	Current density (mA cm^−2^)	Applied charge (F mol^−1^)	Phenolic OH (mmol g^−1^)	Aliphatic OH (mmol g^−1^)	Ratio aliph. OH/phen. OH
Zn	30	2	6.630	3.401	0.513
Zn	30	8	6.236	2.730	0.438
Zn	60	8	8.775	4.362	0.497
GC	60	8	8.234	0.513	0.067

Finally, it should be noted that in comparison higher weight-average molecular weights *M*_W_ up to 17 000 g mol^−1^ of polyvanillin synthesized at a Pb cathode with a divanillin concentration of 0.175 M were reported in the preliminary feasibility study by Amarasekara *et al.*, which could not be reproduced.^23^ However, exact current densities, SEC calibration standards and columns were not stated and DMF was used as solvent for SEC analysis. Therefore, the SEC molecular weight data of polyvanillin is hardly comparable. However, the published ^1^H-NMR spectra of the polyvanillin sample showed unreacted aldehyde end groups and a sharp methoxy group peak indicating not full conversion of divanillin and existence of a high amount of a very low molecular fraction. Therefore, relatively higher molecular weights should have been achieved in this presented study.

## 4 Conclusions

In conclusion, vanillin was successfully exploited for the electrochemical production of biobased polymers (polyvanillin) and polymer building blocks (hydrovanilloin). The production of these compounds was realized in an H-type cell setup by reductive coupling *via* pinacolization of vanillin and divanillin to hydrovanilloin and polyvanillin, respectively, and dependencies of cathode material, current density and applied charge on their formation were systematically investigated. Selected viable cathode materials for the electrochemical coupling steps exhibiting high HER overpotentials (Pb, GC and Zn) were screened by LSV for their activity. The screening was followed by bulk electrolysis experiments of vanillin gaining insights into conversion, product distributions and FEs under various reaction conditions. Thereby, Zn was revealed as stable, sustainable and excellent replacement for the commonly used toxic cathode material Pb enhancing the selectivity for hydrovanilloin formation to almost 100% up to high current densities of 100 mA cm^−2^ at reasonable FEs of 55–85%. Further, impact parameters on structural features of polyvanillin, generated in the divanillin bulk electrolysis experiments, were examined by SEC, 2D-NMR (HSQC, ^13^C/^1^H) and ^31^P-NMR after *in situ* labeling with Cl-TMDP. SEC analyses revealed weight-average molecular weights *M*_W_ up to 3400 g mol^−1^ (*vs.* pullulan standard) at low polydispersities. At Zn cathodes the molecular weights and their distributions of polyvanillin were observed to be independent on current density (15–60 mA cm^−2^) and divanillin concentration (0.1–0.3 M), whereas for Pb and GC cathodes molecular weights decreased at higher current densities. Structural features such as unreacted aldehyde groups, pinacol groups, terminal alcohol groups and stilbene-like double bound systems were identified by NMR and their dependency on reaction conditions discussed.

For further scalability of these promising electrochemical processes a transfer to an electrochemical flow reactor is required, as batch cells are limited in production rate due to low electrode area to electrolyte ratios and may suffer from poor undefined hydrodynamics.^54^ Thus, the transfer to an electrochemical flow reactor of these processes including a detailed investigation of the mass transport behavior will be addressed in future investigations overcoming the mentioned limitations of batch cells.

## Author contributions

Conceptualization, R. Kunkel, V. M. Schmidt; data curation, R. Kunkel and D. Schmiedl; formal analysis, R. Kunkel and D. Schmiedl; funding acquisition, D. Schmiedl, C. Cremers and J. Tübke; investigation, R. Kunkel, D. Müller and D. Schmiedl; methodology, R. Kunkel and D. Schmiedl; project administration, C. Cremers, D. Schmiedl and J. Tübke; supervision, V. M. Schmidt and J. Tübke; visualization, R. Kunkel; writing – original draft, R. Kunkel; writing – review & editing, R. Kunkel, V. M. Schmidt, C. Cremers and D. Schmiedl.

## Conflicts of interest

There are no conflicts to declare.

## Supplementary Material

RA-011-D1RA00649E-s001
