# Supplementary material for: Electrochemical synthesis of biobased polymers and polymer building blocks from vanillin
Source: RSC Adv. 2021 Mar 1;11(15):8970–85. doi: 10.1039/d1ra00649e (PMC8695360; doi:10.1039/d1ra00649e)
Supplement: RA-011-D1RA00649E-s001 [file RA-011-D1RA00649E-s001.pdf]

Electronic Supplementary Material (ESI) for RSC Advances.
This journal is © The Royal Society of Chemistry 2021

Electronic Supplementary Information (ESI)

Electrochemical Synthesis of Biobased Polymers and Polymer Building Blocks from Vanillin

Robin Kunkel[1, *], Volkmar M. Schmidt[2], Carsten Cremers[1], Dominik Müller[1], Detlef Schmiedl[3], Jens Tübke[1]

[1]Fraunhofer Institute for Chemical Technology ICT, Department of Applied Electrochemistry, Joseph-von-Fraunhofer-Str. 7, D-76327 Pfinztal, Germany

[2]Mannheim University of Applied Sciences, Institute of Chemical Process Engineering, Paul-Wittsack-Str. 10, D-68163 Mannheim, Germany

[3]Fraunhofer Institute for Chemical Technology ICT, Department of Environmental Engineering, Joseph-von-Fraunhofer-Str. 7, D-76327 Pfinztal, Germany

[*] Corresponding author: robin.kunkel@ict.fraunhofer.de

1. Product analysis of the divanillin synthesis

The product structure was confirmed by LC-DAD-MS, elemental analysis and FT-IR. LC-DAD-MS analysis of first batch synthesis of divanillin is shown in Fig. S1. First peak was assigned to remaining vanillin (4.9 wt.-% determined by GC-FID) and second peak was assigned to divanillin.

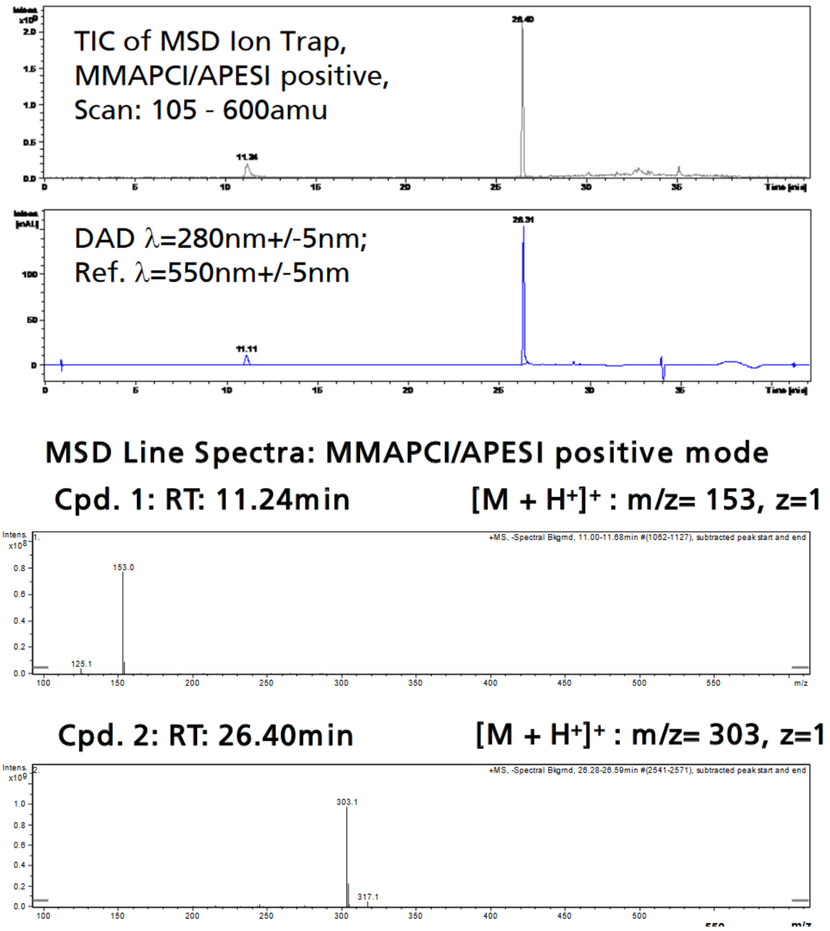

Fig. S1 LC-DAD-MS spectra of divanillin. Set-up - Kinetex C8, 2.6 µm, 100 Å, 3 mm x 100 mm
A: Acetic acid-0.05%; B: Methanol; Gradient, 0-42 min, 0.5 ml min^{-1}, T: 45 °C; MMAPCI/APESI pos.

Elemental Analysis (CHO) for divanillin (Found: C, 62.6; H, 5; O, 32.3. Calc. for $C_{16}H_{14}O_6$: C, 63.6; H, 4.7; O, 31.8%).

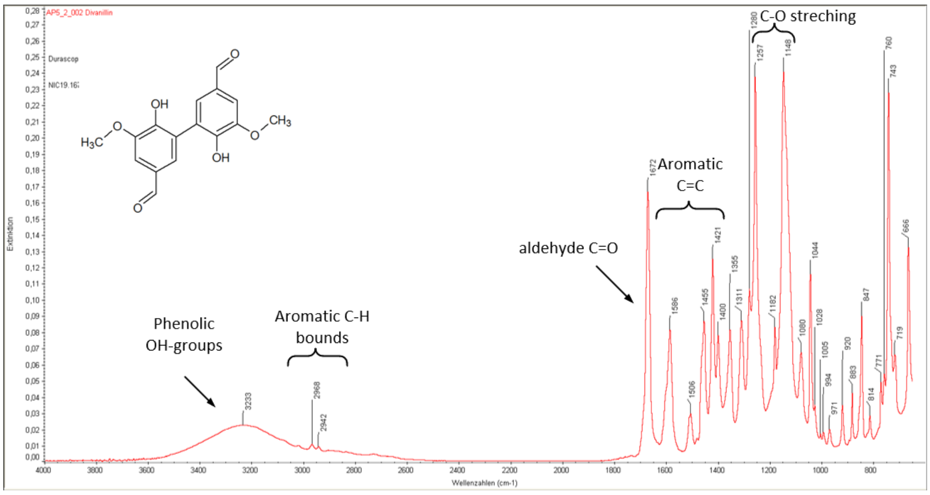

Fig. S2 FT-IR spectrum of divanillin, ATR-mode.

2. Electrochemical reduction of vanillin at GC

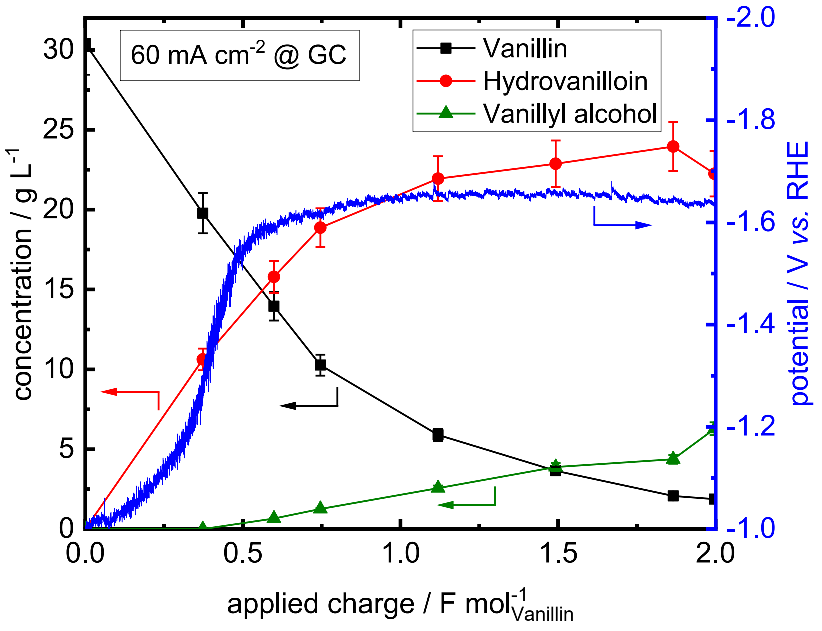

Fig. S3 Potential and concentration course of the electrochemical vanillin reduction at GC at 60 mA cm^{-2}. Analysis was carried out by capillary electrophoreses coupled with UV-Vis detection.

3. Isolation and identification of the product of the electrochemical vanillin reduction

Table S1 *Isolated product yields after an applied charge of 2 F mol^{-1} at different current densities and at different cathode materials.*[a]

Cathode material	Current density (mA cm-2)	Isolated product yield[b] after an applied charge of 2 F mol^{-1} (%)
Zn	30	72.4
	60	64.0
	100	48.6
Pb	30	71.7
	60	69.8
	100	57.7
GC	30	69.1
	60	62.4
	100	50.0

[a]Electrolysis was performed under following conditions - catholyte: 0.2 M Vanillin in 1 M sodium hydroxide, anolyte: 1 M sodium hydroxide, room temperature, catholyte stirred at 250 rpm. [b]Isolation of the product was carried out by titrating the catholyte to pH = 2 with 1 M HCl followed by filtration, washing and drying.

The isolated product was identified by HPLC-DAD-MSD, FT-IR and ^{1}H-, ^{13}C- and 2D-NMR (HSQC, ^{13}C/^{1}H) as pure hydrovanilloin. Exemplary spectra of isolated hydrovanilloin synthesized at a Zn cathode at 30 mA cm^{-2} with an applied charge of 2 F mol^{-1} are shown in Fig.S4-S6. In the HPLC-DAD-MSD spectra, however, two product peaks were observed. The first peak was assigned to hydrovanilloin showing a molecule signal with a monoisotopic mass of 306 g mol^{-1} and a signal with a monoisotopic mass of 288 g mol^{-1} due to water fragmentation from the pinacol group. The second peak solely shows the water fragmentated signal with a monoisotopic mass of 288 g mol^{-1}, which would correspond to 1,2-bis(4-hydroxy-3-methoxyphenyl)ethanon being in keto-enol tautomeric equilibrium with 1,2-bis(4-hydroxy-3-methoxyphenyl)hydroxyethene. We suspect that dissolution of the isolated product in methanol for HPLC-DAD-MSD analysis leads to the water fragmentation. The ^{1}H-, ^{13}C- and 2D-NMR (HSQC, ^{13}C/^{1}H) spectra of the same product reveal that peak 2 of the HPLC-DAD-MSD spectra is an artefact, as no water fragmentation is observed. Since the reductive electrochemical coupling of vanillin by pinacolization is a stereoselective reaction, two forms, namely the DL-form and the meso-form, are possible. The ratio between these two forms can by calculated by the methoxy groups signals or the Cα-Hα / Cα′-Hα′ signals in the aliphatic region of hydrovanilloin in the ^{1}H-NMR spectra. The methoxy group signals were assigned to 3.67 ppm (meso-form) and 3.61 ppm (DL-form) and the Cα-Hα / Cα′-Hα′signals to 4.47 ppm (meso-form) and 4.41 (DL-form). A meso- to DL-form ratio of 78% is calculated.

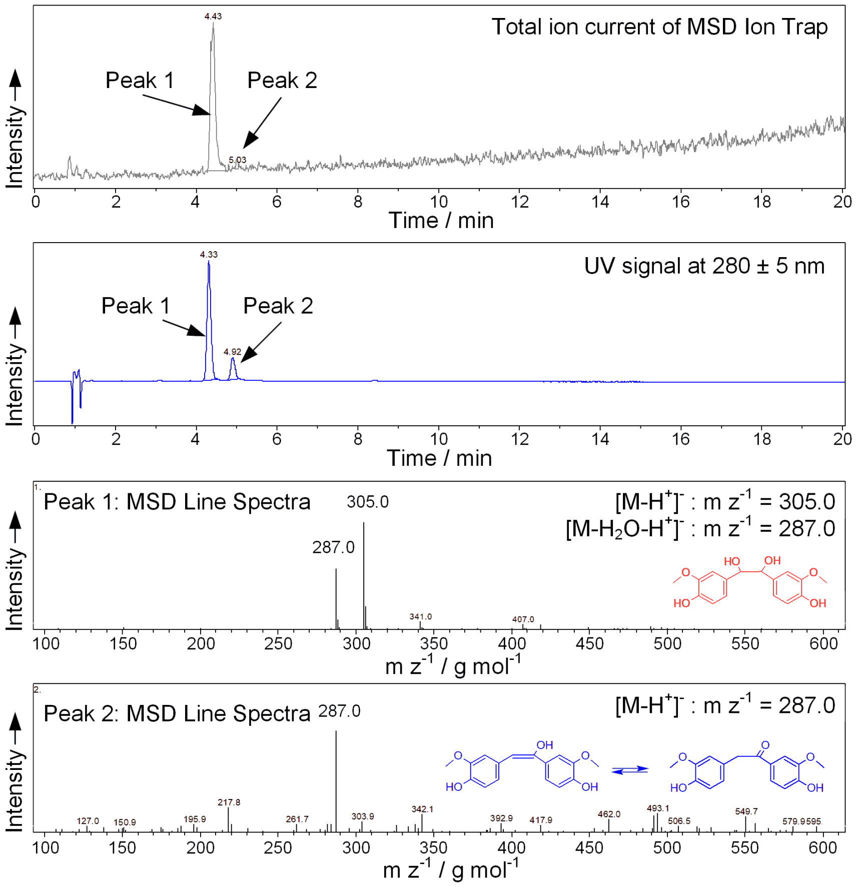

Fig S4 HPLC-UV-MSD spectra of the isolated product dissolved in MeOH for analysis. (Vanillin concentration 0.2 M in 1 M sodium hydroxide solution, Zn cathode, 30 mA cm^{-2}, 2 F mol^{-1}, catholyte stirred at 250 rpm, RT)

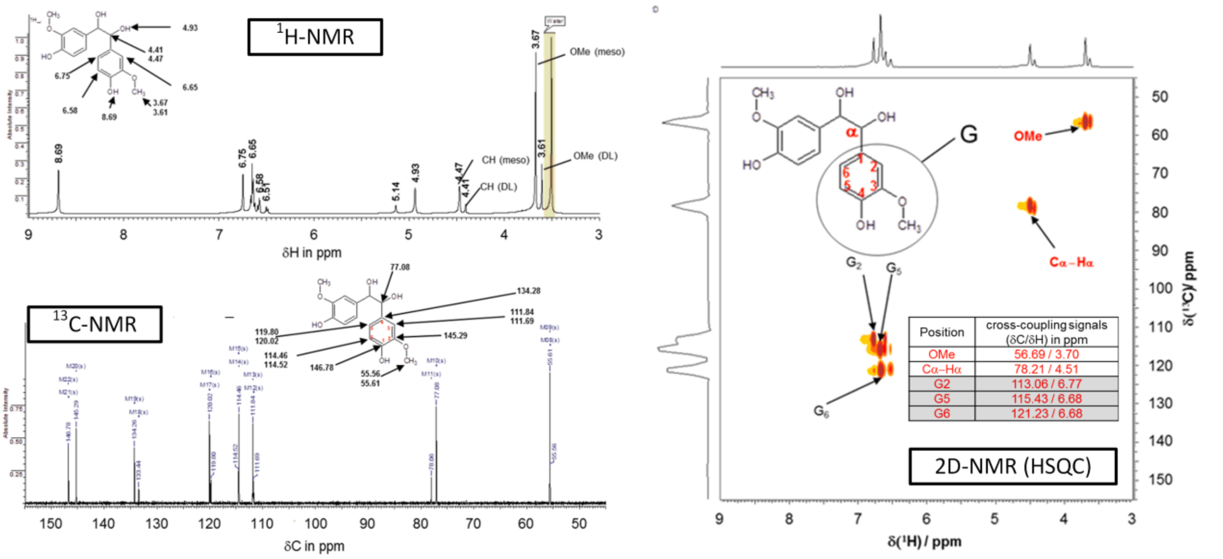

Fig S5 ^{1}H-, ^{13}C- and 2D-NMR (HSQC, $^{13}C/^{1}H$) spectra of the isolated product dissolved in DMSO-d_6 recorded on a Bruker AVANCE spectrometer operating at 500 MHz. (Vanillin concentration 0.2 M in 1 M sodium hydroxide solution, Zn cathode, 30 mA cm^{-2}, 2 F mol^{-1}, catholyte stirred at 250 rpm, RT)

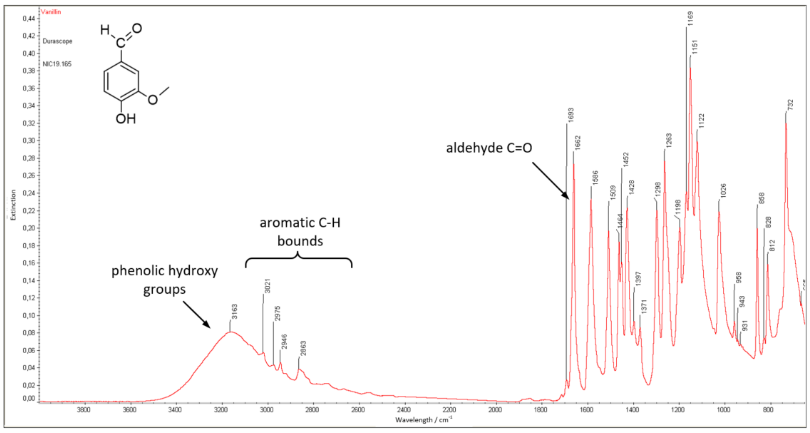

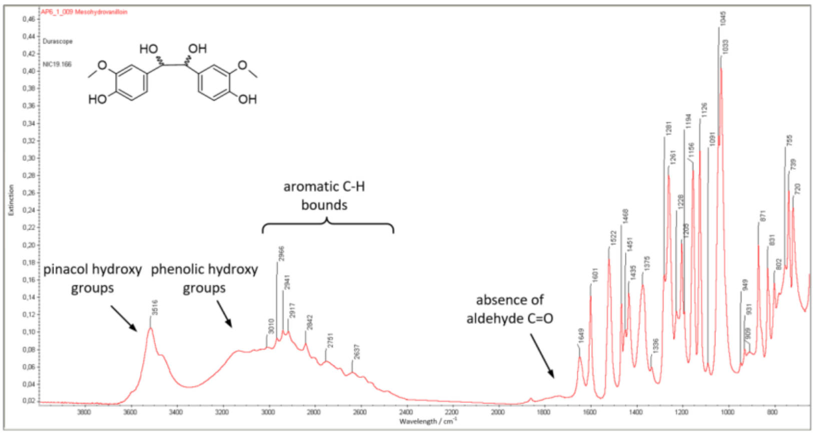

Fig. S6 FT-IR spectra of vanillin and hydrovanilloin, ATR-mode.

4. Electrochemical synthesis of polyvanillin

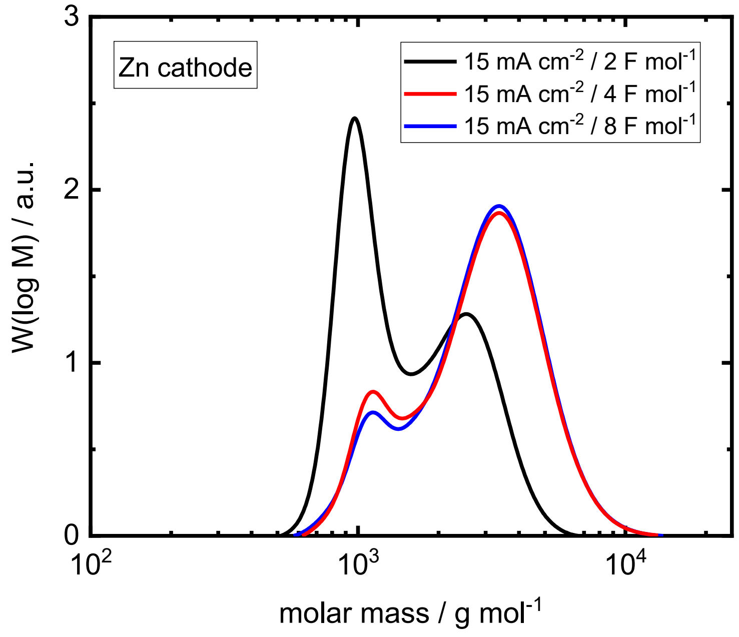

Fig. S7 Molecular weight distribution of isolated polyvanillin determined by SEC (calibrated versus pullulan standard) after different amount of applied charges at a current density of 15 mA cm^{-2} (run 1-3).

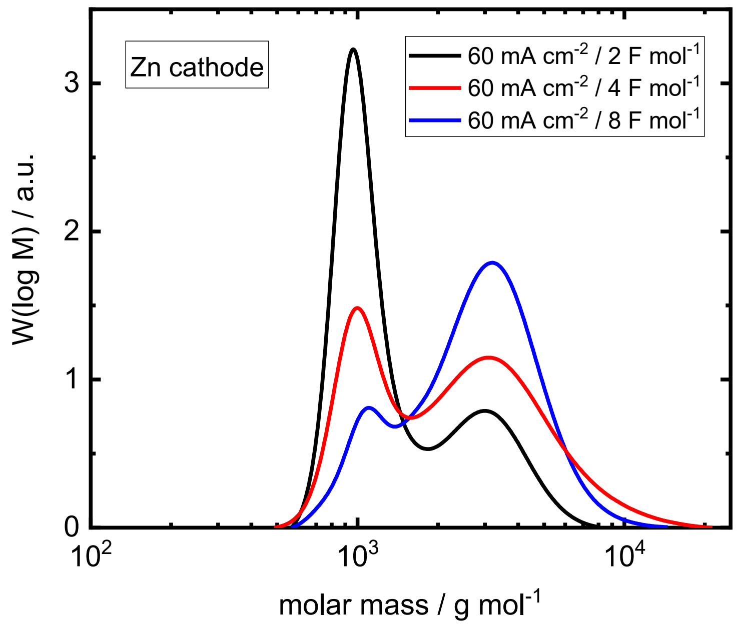

Fig. S8 Molecular weight distribution of isolated polyvanillin determined by SEC (calibrated versus pullulan standard) after different amount of applied charges at a current density of 60 mA cm-2 (run 7-9).

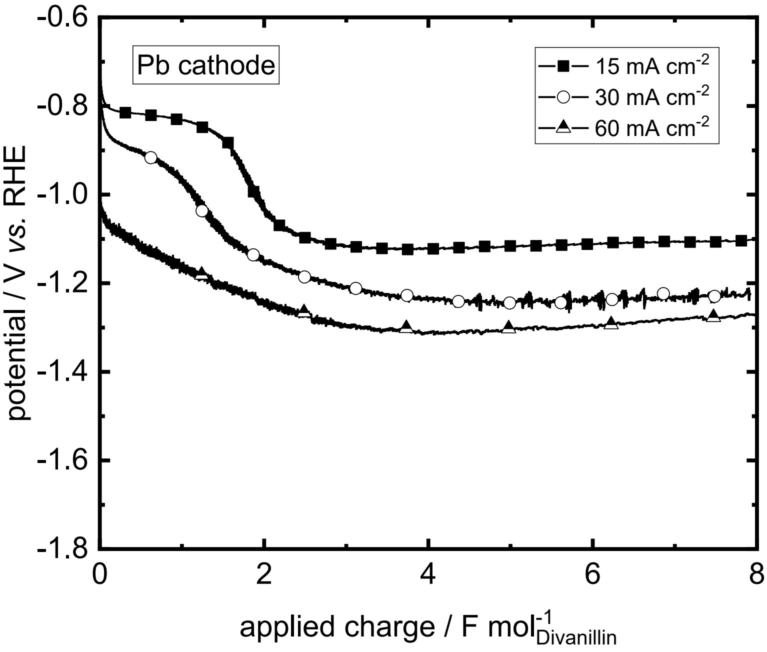

Fig. S9 Potential-charge plots of the electrochemical divanillin reduction at a Pb cathode (5 cm^2). Electrolysis was performed under following conditions – anode: Ni-foam, catholyte: 0.1 M divanillin in 1 M sodium hydroxide (first batch of divanillin), anolyte: 1 M sodium hydroxide, room temperature, catholyte stirred at 250 rpm.

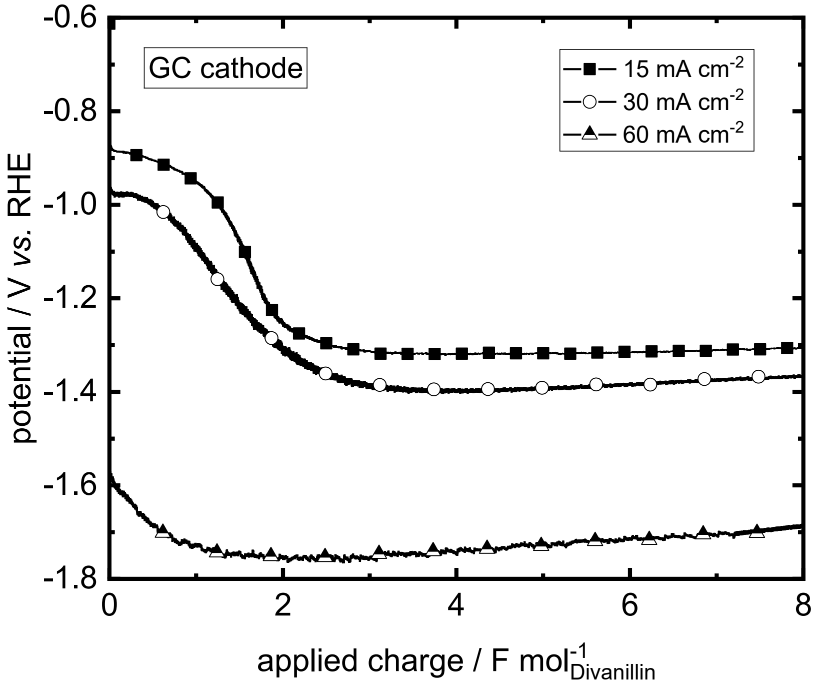

Fig S10 Potential-charge plots of the electrochemical divanillin reduction at a GC cathode (5 cm^2). Electrolysis was performed under following conditions – anode: Ni-foam, catholyte: 0.1 M divanillin in 1 M sodium hydroxide (first batch of divanillin), anolyte: 1 M sodium hydroxide, room temperature, catholyte stirred at 250 rpm.

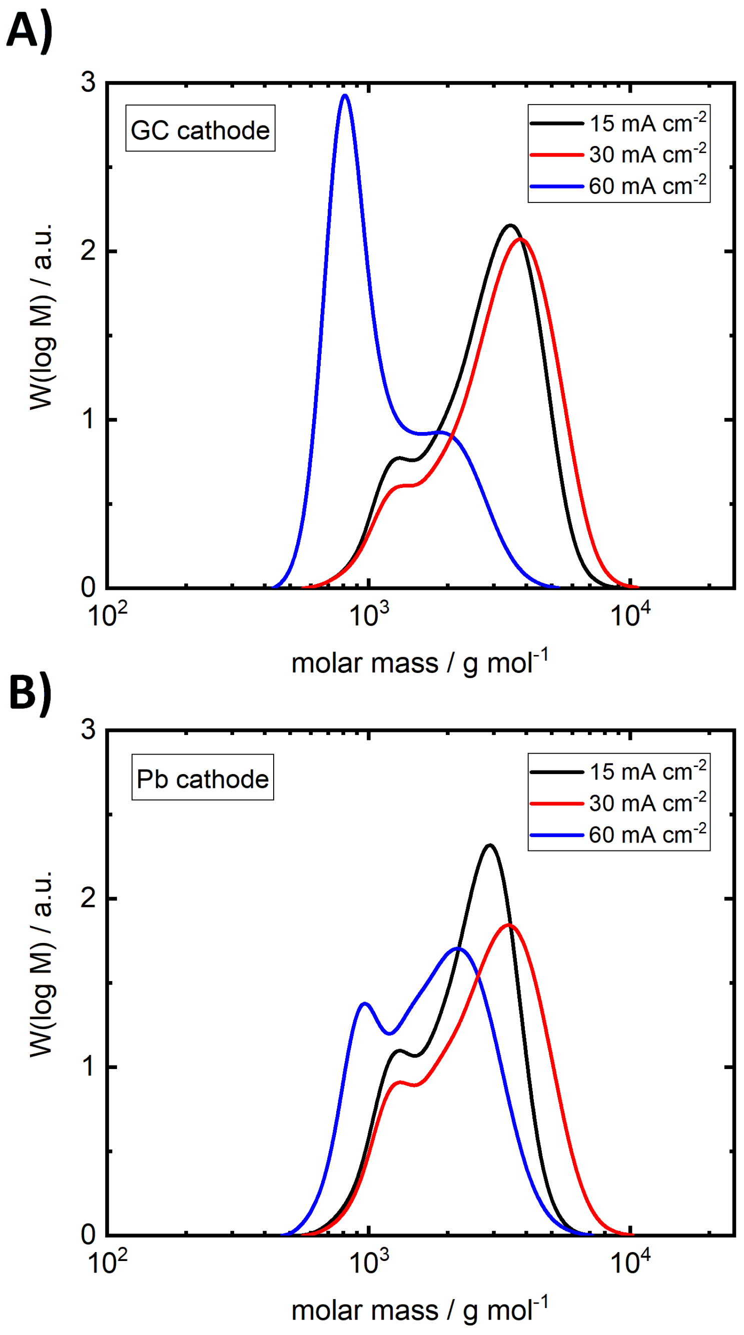

Fig S11 MWDs of isolated polyvanillin synthesized at a glassy carbon cathode (A) and a Pb cathode (B). Electrolysis was performed under following conditions – applied charge: 8 F mol^{-1}, catholyte: 0.1 M divanillin in 1 M sodium hydroxide (first batch of divanillin), anolyte: 1 M sodium hydroxide, room temperature, catholyte stirred at 250 rpm, isolated products were analyzed by SEC. SEC was calibrated versus pullulan standard.

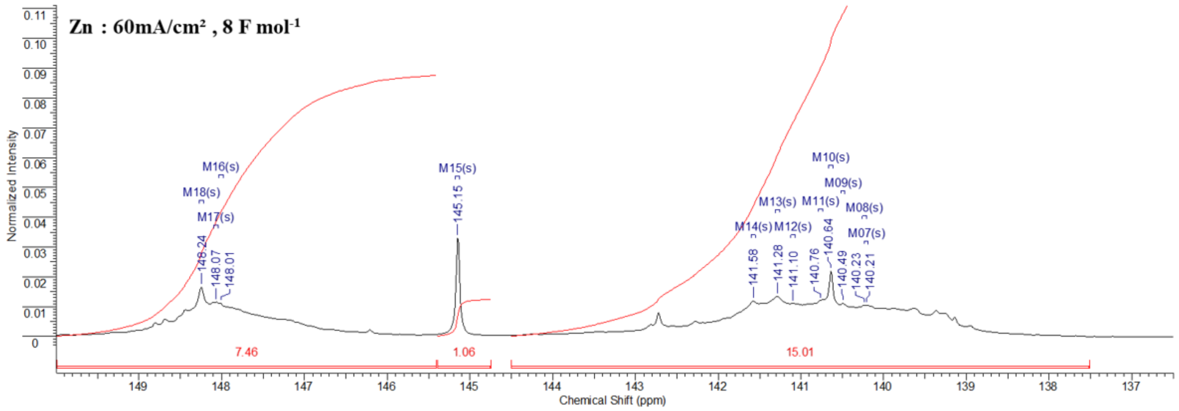

Fig. S12 ^{31}P-NMR analysis of the polyvanillin sample generated by electrochemical pinacolization of 5,5′-bis-vanillin at a Zn-cathode at a current density of 60 mA cm^{-2} and an applied charge of 8 F mol^{-1}, after in-situ labelling with 2-Chloro-4,4,5,5-tetramethyl-1,3,2-dioxaphospholane (Cl-TMDP) in pyridine / $CDCl_3$ = 1.6 / 1 (v / v). Chemical shifts are reported relative to the sharp signal (132.2 ppm) originating from the reaction between traces of H_2O and Cl-TMDP. Internal standard: Cyclohexanol (peak: 145.15 ppm); integration region for aliphatic hydroxy groups: 150.0-145.4 ppm and for total phenolic hydroxy groups: 144.5 – 137.5 ppm.
